# A tyrosine aminotransferase involved in rosmarinic acid biosynthesis in *Prunella vulgaris* L

**DOI:** 10.1038/s41598-017-05290-4

**Published:** 2017-07-07

**Authors:** Mei Ru, Kunru Wang, Zhenqing Bai, Liang Peng, Shaoxuan He, Yong Wang, Zongsuo Liang

**Affiliations:** 1 Institute of Soil and Water Conservation, Chinese Academy of Sciences&Ministry of Water Resources, Yangling, 712100 P.R. China; 2 College of Pharmacy, Shannxi University of Chinese Medicine, Xi’an, 710000 P.R. China; 3 Ecological Environmental Monitoring Station, Environmental Protection Agency, Dazu, 402360 P.R. China; 4 College of Life Sciences, Zhejiang Sci-Tech University, Hangzhou, 310000 P.R. China

## Abstract

Rosmarinic acid (RA) and its derivants are medicinal compounds that comprise the active components of several therapeutics. We isolated and characterised a tyrosine aminotransferase of *Prunella vulgaris* (PvTAT). Deduced PvTAT was markedly homologous to other known/putative plant TATs. Cytoplasmic localisation of PvTAT was observed in tobacco protoplasts. Recombinantly expressed and purified PvTAT had substrates preference for L-tyrosine and phenylpyruvate, with apparent *K*_m_ of 0.40 and 0.48 mM, and favoured the conversion of tyrosine to 4-hydroxyphenylpyruvate. *In vivo* activity was confirmed by functional restoration of the *Escherichia coli* tyrosine auxotrophic mutant DL39. *Agrobacterium rhizogenes*-mediated antisense/sense expression of *PvTAT* in hairy roots was used to evaluate the contribution of PvTAT to RA synthesis. *PvTAT* were reduced by 46–95% and RA were decreased by 36–91% with low catalytic activity in antisense transgenic hairy root lines; furthermore, *PvTAT* were increased 0.77–2.6-fold with increased 1.3–1.8-fold RA and strong catalytic activity in sense transgenic hairy root lines compared with wild-type counterparts. The comprehensive physiological and catalytic evidence fills in the gap in RA-producing plants which didn’t provide evidence for TAT expression and catalytic activities *in vitro* and *in vivo*. That also highlights RA biosynthesis pathway in *P*. *vulgaris* and provides useful information to engineer natural products.

## Introduction

The popular medicinal plant, *Prunella vulgaris*, known as ‘heal-all’ or ‘self-heal’, has therapeutical applications in alleviating sore throats, reducing fever, and accelerating wound healing^1^. Rosmarinic acid (RA), a phenolic ester, is a prominent secondary metabolite member of the Lamiaceae and Boraginaceae. This compound and its derivates show many notable biological and pharmacological activities, such as anti-colitic^2^, antioxidant^3, 4^, anti-inflammatory^5, 6^, anti-leukemic^7^, and anticancer activities^8, 9^, as well as neuroprotective activity^10, 11^, which has led to their pharmaceutical and analytical development, and examination in clinical studies. *In vitro* culture systems producing RA, including callus, suspension cell cultures, hairy root cultures, and shoot cultures, have been successfully established from many plant species, such as *Coleus blumei* ^12, 13^, *Anchusa officinalis* ^14^, *Salvia miltiorrhiza* ^15, 16^, *Salvia officinalis* ^17^, *Agastache rugosa* ^18^, *Ocimum basilicum* ^19, 20^, and *Coleus forskohlii* ^21^. RA levels in some *in vitro* culture systems are higher than in the parent plant. Engineered production of RA and its analogues have also been achieved in an *Escherichia coli* culture system^22–24^.

**Figure 1:**
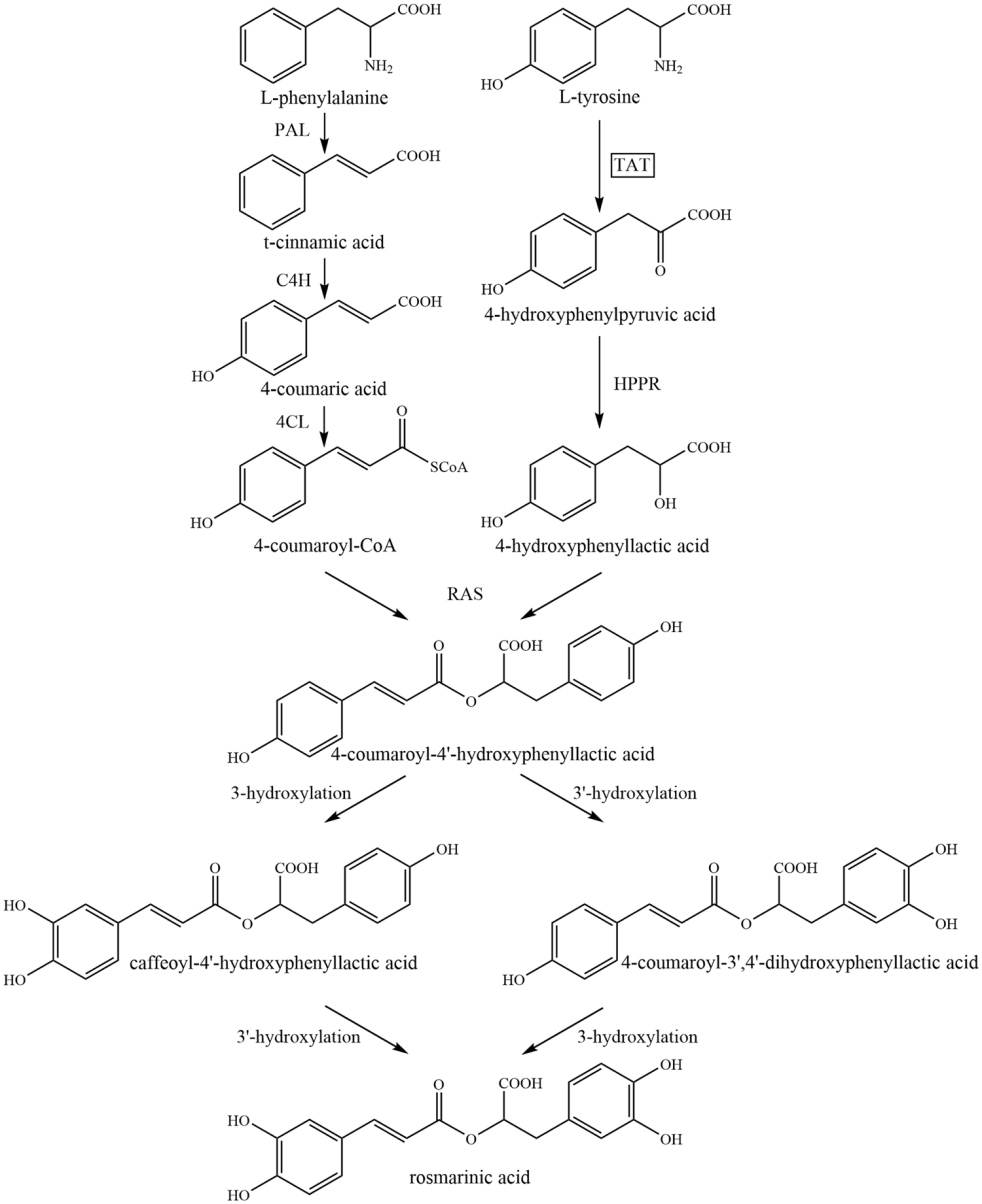
Biosynthetic pathway leading to rosmarinic acid formation in *Coleus blumei* ^29, 30^. PAL: phenylalanine ammonia lyase; C4H: cinnamic acid 4-hydroxylase; 4CL: 4-coumaric acid CoA-ligase; TAT: tyrosine aminotransferase; HPPR: hydroxyphenylpyruvate reductase; RAS: rosmarinic acid synthase (4-coumaroyl-CoA:4′-hydroxyphenyllactic acid 4-coumaroyltransferase); 3- and 3′-hydroxylation can be catalyzed by cytochrome P450-monooxygenase^37^.

Feeding radioactively labeled amino acids to *Mentha* plants and *Coleus* suspension cell cultures have revealed that phenylalanine (Phe) and tyrosine (Tyr) are the only respective amino acid precursors of the caffeoyl moiety and 3,4-hydroxyphenyllactic acid (DHPL) moiety of RA^25–27^. Tyrosine aminotransferase (TAT) was confirmed as the entry point enzyme of the tyrosine-derived pathway in RA biosynthesis^28^. Petersen, *et al*.^29^ proposed that RA biosynthesis in *C*. *blumei* involves both the phenylpropanoid pathway (for the caffeic acid moiety) and the tyrosine-derived pathway (for the DHPL moiety) (Fig. 1). Enzymes and genes of RA biosynthesis are well investigated in *C*. *blumei*, *S*. *miltiorrhiza*, *Melissa officinalis*, *Lithospermum erythrorhizon*, and other members of the Lamiaceae and Boraginaceae families^30^. TAT reversibly catalyses Tyr to 4-hydroxyphenylpyruvate (4-HPP), which is the substrate for pathways producing plastoquinone^31, 32^, tocopherols^33^, phenolic acid^14, 28^, and benzylisoquinoline alkaloid (BIA)^34^ in plants, although tyrosine is synthesised from 4-HPP by TAT in bacteria. These Tyr-derived plant metabolites have a noteworthy structural complexity and a variety of pharmacological and biological activities, making them effective nutritional compounds and pharmaceutical drugs. RA is a major component of phenolic acids in *Prunella vulgaris* and controls the quality of this medicinal plant according to National Chinese Pharmacopeia^35^. Kim, *et al*.^36^ cloned the genes of enzymes involved in the phenylpropanoid branched-chain of the RA biosynthetic pathway in *P*. *vulgaris*, however, no biochemical or physiological information has been supported about these enzymes. Moreover, little information about enzymes involved in tyrosine-derived pathway is available in *P*. *vulgaris*.

Although the biochemical and structural characterisations of TATs in *E*. *coli* ^38^, *Trypanosoma cruzi* ^39^, mouse^40^, *Leishmania infantum* ^41^, and *Homo sapiens* (Protein Data Bank code, 3dyd) are well established, considerably less information about these enzymes is available in plants. Three activities of TAT have been extensively purified and characterised from *A*. *officinalis* cell cultures^14^. TAT has been implicated as the initial enzyme in tocopherol biosynthesis in Arabidopsis plants^33, 42, 43^, in aromatic volatiles biosynthesis of *Cucumis melo* fruits^44^, in BIA biosynthesis of *Papaver somniferum* ^34^, in an alternate pathway for phenylalanine biosynthesis^45^. Aminotransferase activity has been shown for Arabidopsis TATs^33, 42, 46–48^, *C*. *melo* ArAT1^44, 45^, *P*. *somniferum* TAT^34^, rose PyAAAT3^49^, petunia PhPPY-AT^45^, *Atropa belladonna* ArAT4^50^, and *Ephedra sinica* AroAT1^51^ by activity measurements of the isolated proteins expressed in bacterial systems and/or complementation of the auxotrophic *E*. *coli* strain DL39. Despite enhanced RA accumulation has been demonstrated by overexpressing *TAT* in *S*. *miltiorrhiza* hairy roots and transgenic *P*. *frutescens* plants^52, 53^. However, biochemical confirmation of their enzymatic activity has not been shown in major RA-producing plants. In plants, tocopherols and RA have been defined roles in free radical scavenging and in defence against abiotic and biotic stresses^11, 54^, and BIA are used by humans as stimulants, narcotics, and therapeutic agents^50, 55^. These natural products increase plant fitness and are also linked to potential benefits for human health.

Although much work has been done to identify the genes responsible for RA metabolism, the enzymes involved in the supply of precursors are still poorly defined in *P*. *vulgaris*. Here, we isolated and characterised tyrosine aminotransferase cDNA (*PvTAT*) using homology-based cloning, determined its subcellular localisation, characterised the heterologously overexpressed protein, and showed its functional complementation for tyrosine auxotrophic mutant DL39. Moreover, antisense/sense-based expression showed PvTAT is involved in RA biosynthesis *in planta*.

## Results

### Identification of a tyrosine aminotransferase as a candidate rosmarinic acid biosynthetic enzyme

A deep transcriptome database was generated by 454 GS-FLX Titanium pyrosequencing using a cDNA library prepared from *P*. *vulgaris* root (http://www.ncbi.nlm.nih.gov/sra/SRX130243). In search of the transcriptome database of *P*. *vulgaris* using *S*. *miltiorrhiza TAT* (KM575934.1) and *P*. *frutescens TAT* (AJ458993.1) as the query sequence revealed the presence of highly homologous unigenes (similarity > 80%). We used homologous cloning and obtained a cDNA belonging to the PLP-dependent Asp aminotransferase superfamily (AAT-like proteins) and also belonging to the tyrosine aminotransferase (TAT) subfamily according to the Panther classification system (http://www.pantherdb.org/). The cDNA contained a 1,236-bp ORF (Supplemental Fig. S1, *lane* 1) and encoded a predicted translation product of 411 amino acids with a molecular mass of 45 kDa and an isoelectric point of 5.97. The gene had a sequence of 2,634-bp (Supplemental Fig. S1, *lane* 2) with seven extrons and six introns and was identical to the cDNA except for three nucleotides. These differences did not result in amino acid substitutions.

**Figure 2:**
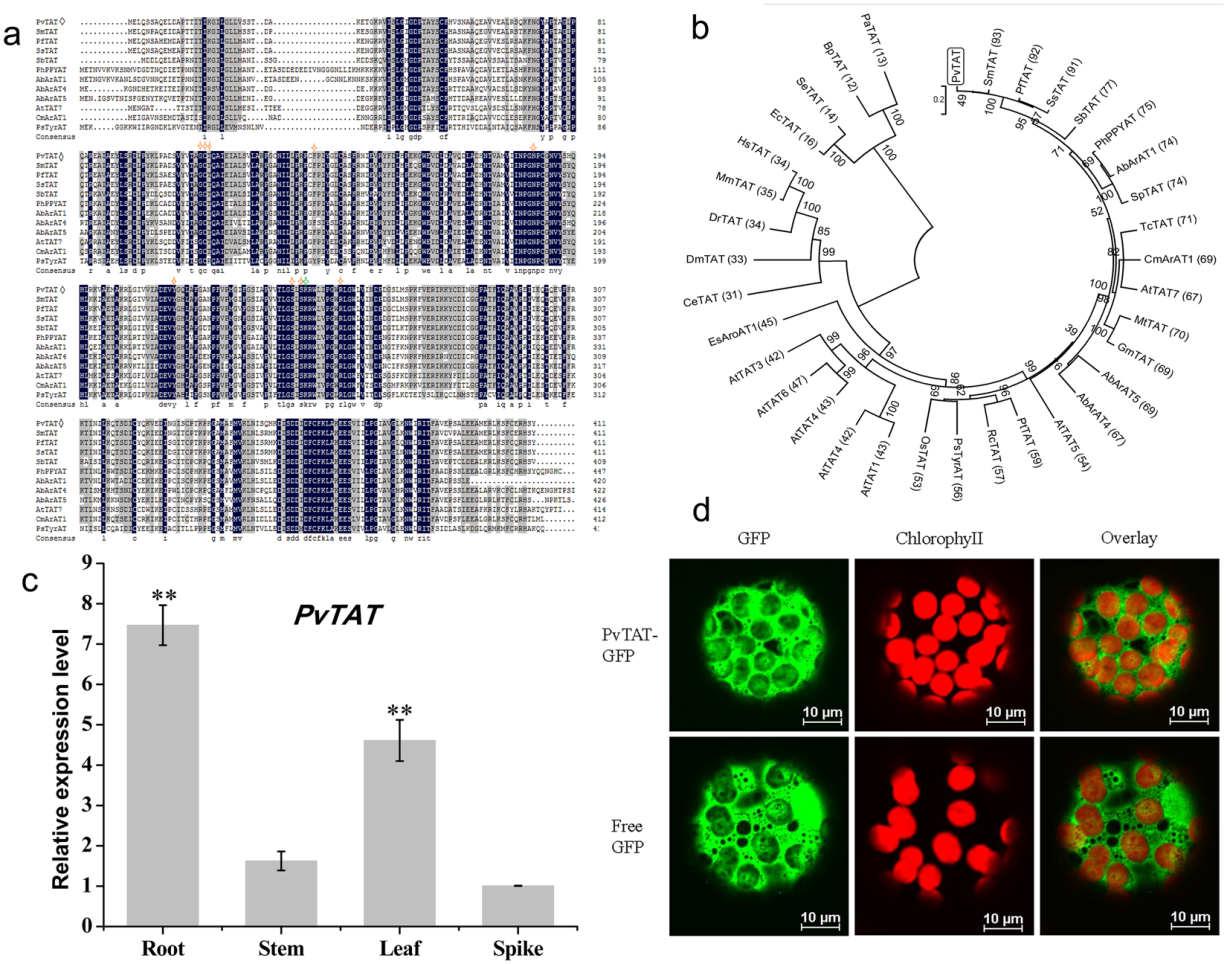
Multiple sequence alignment, phylogenetic tree, expression pattern, and subcellular localisation of *P*. *vulgaris* TAT. (**a**) Amino acid sequence alignment of tyrosine aminotransferase (PvTAT) from *Prunella vulgaris* and selected homologs. The amino acid alignment was generated using DNAMAN 7.0. Identical and similar amino acids are shaded in black and gray, respectively. The catalytic site of the lysine residue is marked with green asterisk. The pyridoxal-5′-phosphate binding sites are marked with orange asterisks. PvTAT is marked with rhombus. Abbreviations are defined in the Table S1. (**b**) Phylogenetic relationship of and related proteins. An unrooted neighbour-joining tree was constructed using MEGA 5.05 software. Numbers refer to the percentage bootstrap test support for each node over 1,000 bootstrap iterations. Numbers in parentheses show the percentage amino acid identity of each protein compared with TAT from *P*. *vulgaris*. The identifiers of the proteins are provided in Supplemental Table S1. (**c**) Relative expression of *PvTAT* in various organs of *P*. *vulgaris*. Gene relative expression was determined by qRT-PCR and is shown relative to the expression level of the housekeeping gene β-actin. Relative expression was calculated using the 2^−ΔΔt^ method.Transcripts of *PvTAT* in spikes were set to 1. Data are means ± SD of three replicates. *P < 0.05 and **P < 0.01 as determined by Tukey HSD test. (**d**) Subcellular localisation of PvTAT. The *PvTAT*-*GFP* or free *GFP* construct was expressed in tobacco protoplasts and the fluorescence was observed under a confocal microscopy. PvTAT-GFP fusion protein localises in the cytoplasm in tobacco protoplasts. Free GFP served as the control. GFP fluorescence is in green and chloroplasts fluorescence is in red.

An amino acid alignment of the predicted PvTAT sequence and selected homologs indicates that each contains a conserved catalytic lysine that corresponds to residue 246 in PvTAT, along with conserved residues required for the binding of the pyridoxal-5′-phosphate cofactor (Fig. 2a). The NCBI Conserved Domain Database (http://www.ncbi.nlm.nih.gov/Structure/cdd/cdd.shtml) structure prediction tool also suggested that the PvTAT candidate possessed several homodimer interfaces. An unrooted neighbour-joining tree representing the phylogenetic relationships between the putative PvTAT and related enzymes is shown in Fig. 2b. PvTAT showed the highest sequence identity (76–93%) with *S*. *miltiorrhiza* SmTAT (ABC60050), *P*. *frutescens* PfTAT (ADO17550.1), *Solenostemon scutellaridoides* SsTAT (CAD30341), and *Scutellaria baicalensis* SbTAT (AIV98132.1) from the same Labiatae family. The PvTAT protein also exhibited considerable sequence identity (65–75%) with *Solanum pennellii* SpTAT (ADZ24702.1), *A*. *belladonna* Ab-ArAT1 (AHN10101.1), *A*. *belladonna* Ab-ArAT5 (AHN10105.1), *Medicago truncatula* MtTAT (AAY85183.1), *Glycine max* GmTAT (AAY21813.1), and *Theobroma cacao* TcTAT (XP_007021573.1). However, it is important to note that there is no biochemical confirmation of the enzymatic activities of these proteins available. In contrast, PvTAT showed 43–74% sequence identity with *P*. *somniferum* PsTAT (ADC33123.1)^34^, Arabidopsis AtTAT1 (AAK82963.1)^42, 43^, AtTAT2 (AF301899_1)^56^, AtTAT5 (NP_198465.3)^46^, AtTAT7 (NP_200208.1)^33, 47^, *C*. *melo* CmArAT1 (ADC45389)^44^, petunia PhPPY-AT (AHA62827.1)^45^, and *E*. *sinica* AroAT1 (AGK24944.1)^51^, which have been shown to function as TATs. Additionally, *A*. *belladonna* Ab-ArAT4 (AHN10104.1) preferentially catalyses the transamination of phenylalanine to phenylpyruvate and had a sequence identity of 66%^50^. Well characterised TATs in manmals showed 30% sequence identity, while there was 12–15% sequence identity with bacterial TATs. All identifies of the proteins used for phylogenetic analysis are provided in Supplemental Table S1.

### Expression analysis of *PvTAT*

We measured the relative transcript expression level of *PvTAT* in different organs (roots, stems, leaves, and spikes) of *P*. *vulgaris* at complete flowering stage by qRT-PCR. *PvTAT* involved in RA biosynthesis was expressed in all the tissues but possessed distinct expression patterns. The highest transcript abundances were observed in root tissues, followed by leaves. The relative expression levels in roots and leaves were 4.6-and 2.8-fold higher than in the stems, while the transcript level in the spikes was set to 1 (Fig. 2c).

### Cytoplasmic localisation of PvTAT

To experimentally determine the subcellular localisation of the PvTAT *in planta*, we expressed a PvTAT-GFP fusion protein in tobacco protoplasts. An engineered green-shifted green fluorescent protein (GFP), by itself or fused in frame to the C-terminus of TAT, was driven by the cauliflower mosaic virus (CaMV) 35S promoter. The resulting constructs or GFP alone were transformed into tobacco leaves. As shown in Fig. 2d, when GFP alone was expressed, fluorescence was observed almost throughout the cytoplasm and nucleus. Protoplasts with PvTAT-GFP exhibited a different pattern, only the cytoplasm spread fluorescence. Together, the difference between the PvTAT-GFP and GFP free fluorescence pattern suggests that PvTAT is localised in the cytoplasm, which is consistent with the prediction made by PSORT Prediction (http://psort.hgc.jp/form.html).

### PvTAT possesses tyrosine aminotransferase activity

To functionally determine the *in vitro* biochemical roles of the putative PvTAT activity, the pET32a-TAT plasmid or empty vector was expressed in the *E*. *coli* strain Rosetta (DE3) and His-Tag purified to near homogeneity (Supplemental Fig. S2, *lane* 3). The purified recombinant TAT showed evidence of tyrosine aminotransferase activity (Supplemental Fig. S3a). Tyrosine aminotransferase activity in the forward direction forming 4-HPP and glutamate was found to be highest at pH 8.0 and 30 °C (Supplemental Fig. S3b,c), a pH optimum similar to that of found for the identified Arabidopsis At5g36160^46^. The substrate specificity of purified recombinant PvTAT was assessed using three aromatic amino acids as amino donors, together with several potential oxoacid acceptors as potential co-substrates. The activity of PvTAT indicates that it could use L-Tyr, L-Phe, and L-tryptophan (L-Trp) to varying degrees as amino donors and use α-ketoglutarate, pyruvate, oxaloacetate, and phenylpyruvate to diverse degrees as amino acceptors. With α-ketoglutarate as the amino acceptor, PvTAT shows higher preference for L-Tyr amino donor with an apparent *K*_m_ of 0.40 mM than for L-Phe (*K*_m_ of 10.2 mM) and L-Trp (*K*_m_ of 8.14 mM) (Table 1; Supplemental Fig. S4a–c). The catalytic efficiency (*k*_cat_/*K*_m_) for L-Tyr was 20- and 23-fold greater than that of L-Phe and L-Trp. With L-Tyr as the amino donor, PvTAT preferentially utilises aromatic oxoacid phenylpyruvate with a *K*_m_ of 3.90 mM as the amino acceptor, other than aliphatic oxoacid (α-ketoglutarate, pyruvate, and oxaloacetate with a *K*_m_ of 10.80, 52.06, and 4.72 mM, respectively) (Table 1; Supplemental Fig. S4d–g). In the reverse direction with L-glutamate (L-Glu) as amino donor, PvTAT favours 4-HPP as oxoacid acceptor with a *K*_m_ of 0.48 mM (Table 1; Supplemental Fig. S4h–j). To confirm TAT enzyme activity, the native enzyme was compared to heat-inactivated enzyme as a negative control. We determined that PvTAT preferentially catalyses the transamination of L-Tyr to form 4-hpp, which is used as substrate in RA biosynthesis. Unexpectedly, PvTAT has a preference for phenylpyruvate as the preferred amino acceptor rather than α-ketoglutarate. It is likely that PvTAT would catalyse the formation of phenylalanine from tyrosine and phenylpyruvate and that 4-HPP would be produced that *in vivo* would be channeled toward RA biosynthesis.

**Table 1:** Kinetic parameters for PvTAT. Data are presented as means ± SD of three replicates.

Variable substrate (Concentration in assay, mM)	Cosubstrate (Concentration in assay, mM)	*V*_max_ (μmol min^−1^ mg^−1^)	*K*_m_ (mM)	*k*_cat_ (s^−1^)	*k*_cat_/*K*_m_ (mM^−1^s^−1^)
L-Tyr (0–4)	α-ketoglutarate (10)	1.74 ± 0.01	0.40 ± 0.05	1.19 ± 0.009	3.03 ± 0.35
L-Phe (0–50)	α-ketoglutarate (10)	2.27 ± 0.03	10.2 ± 0.05	1.55 ± 0.02	0.15 ± 0.04
L-Trp (0–50)	α-ketoglutarate (10)	1.53 ± 0.12	8.14 ± 1.04	1.05 ± 0.08	0.13 ± 0.008
α-ketoglutarate (0–100)	L-Tyr (5.5)	0.92 ± 0.003	10.80 ± 0.19	0.63 ± 0.002	0.06 ± 0.008
Pyruvate (0–200)	L-Tyr (5.5)	1.30 ± 0.02	52.06 ± 1.38	0.89 ± 0.01	0.02 ± 0.003
Oxaloacetate (0–300)	L-Tyr (5.5)	1.06 ± 0.02	4.72 ± 0.05	0.73 ± 0.01	0.15 ± 0.005
Phenylpyruvate (0–20)	L-Tyr (5.5)	0.43 ± 0.014	3.90 ± 0.067	0.30 ± 0.01	0.076 ± 0.001
4-HPP (0–10)	L-Glu (10)	0.18 ± 0.021	0.48 ± 0.004	0.121 ± 0.01	0.295 ± 0.035
Phenylpyruvate (0–4)	L-Glu (10)	0.22 ± 0.009	0.84 ± 0.01	0.15 ± 0.006	0.18 ± 0.009
Oxaloacetate (0–40)	L-Glu (10)	0.16 ± 0.006	1.82 ± 0.03	0.110 ± 0.004	0.060 ± 0.004

The *K*_m_ and *V*_max_ values were calculated from the Michaelis-Menten equation using a non-linear regression method with curveexpert1.4.

Note: For the calculation of *k*_cat_, a molecular weight of 65 kDa was assumed for the His-tagged TAT protein. And *k*_cat_ was calculated by dividing *V*_max_ by E_t_ (the number [pmol] of enzymes in each assay). The assay measured the production of 4-hydroxyphenylpyruvate at 331 nm, phenylpyruvate at 320 nm, and indole-3-pyruvate at 328 nm corresponding to the substrates L-tyrosine, L-phenylalanine, and L-tryptophan. For the reverse direction, the assay measured the production of NADH at 340 nm.

### TAT functionally complements the *E*. *coli* tyrosine auxotrophic mutant DL39

**Figure 3:**
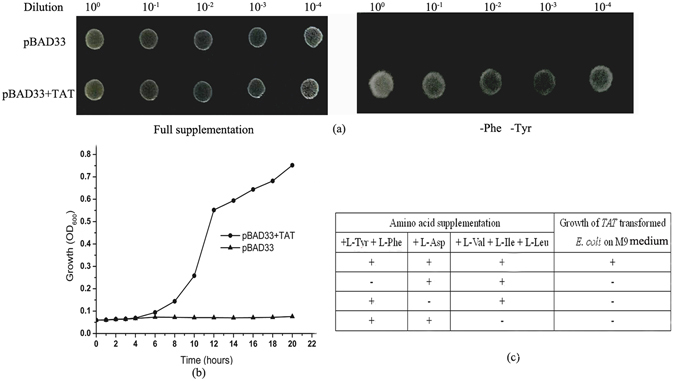
PvTAT complementation of the tyrosine-auxotrophic *E*. *coli* mutant DL39. PvTAT was cloned into the expression vector pBAD33 with an arabinose promoter. The recombinant pBAD33-TAT plasmid or empty plasmid was transformed into the *E*. *coli* triple mutant DL39. The bacteria were grown to an OD of 1.0 measured at 600 nm, the strains were serially diluted to 10^−1^, 10^−2^, 10^−3^, and 10^−4^ using 0.85% (w/v) saline and 3 μl was replica plated on medium with or without L-tyrosine and L-phenylalanine supplemented with 0.2% (w/v) arabinose. (**a**) All transformants could grow on solid M9 medium with full supplementation, but only the plasmids expressing PvTAT could grow on medium free of L-tyrosine and L-phenylalanine. (**b**) Within 20 hours, the growth curve of the DL39 strain transformed with either pBAD33 (triangle) or pBAD33+TAT (circle) in liquid M9 medium lacking L-tyrosine and L-phenylalanine supplemented with 0.2% (w/v) arabinose. (**c**) The summary of the functional complementation assay of the plant enzyme ability to complement the *E*. *coli* triple mutant on M9 medium.

To examine PvTAT activity *in vivo*, the recombinant pBAD33-TAT plasmid or pBAD33^57^ was transformed into the *E*. *coli* triple mutant DL39^58^ for an initial functional complementation assay. This strain has mutations in tyrB (tyrosine aminotransferase), aspC (aspartate aminotransferase), and ilvE (branched-chain aminotransferase), leading to auxotrophy for tyrosine, phenylalanine, aspartate, valine, isoleucine and, leucine. As shown in Fig. 3a, all transformants could grow in M9 medium supplemented with tyrosine, phenylalanine, leucine, isoleucine and valine, whereas only clones transformed with PvTAT were able to grow on M9 medium devoid of tyrosine and phenylalanine. Figure 3b displays the growth curve of the *E*. *coli* mutant transformed with the native plasmid or the plasmid expressing PvTAT. Bacteria transformed with PvTAT grew continuously, while the bacteria transformed with the native plasmid showed little growth over 20 hours. The complementation was only specific to the tyrosine and phenylalanine auxotrophy, because growth did not occur without aspartate or the branched chain amino acids leucine, isoleucine and valine in combination (Fig. 3c). These results show that the putative PvTAT functionally complements the auxotrophic *E*. *coli* mutant and does not participate in the anabolic pathways for aspartate, leucine, isoleucine, and valine.

### PvTAT is involved in RA synthesis in *P*. *vulgaris*

**Figure 4:**
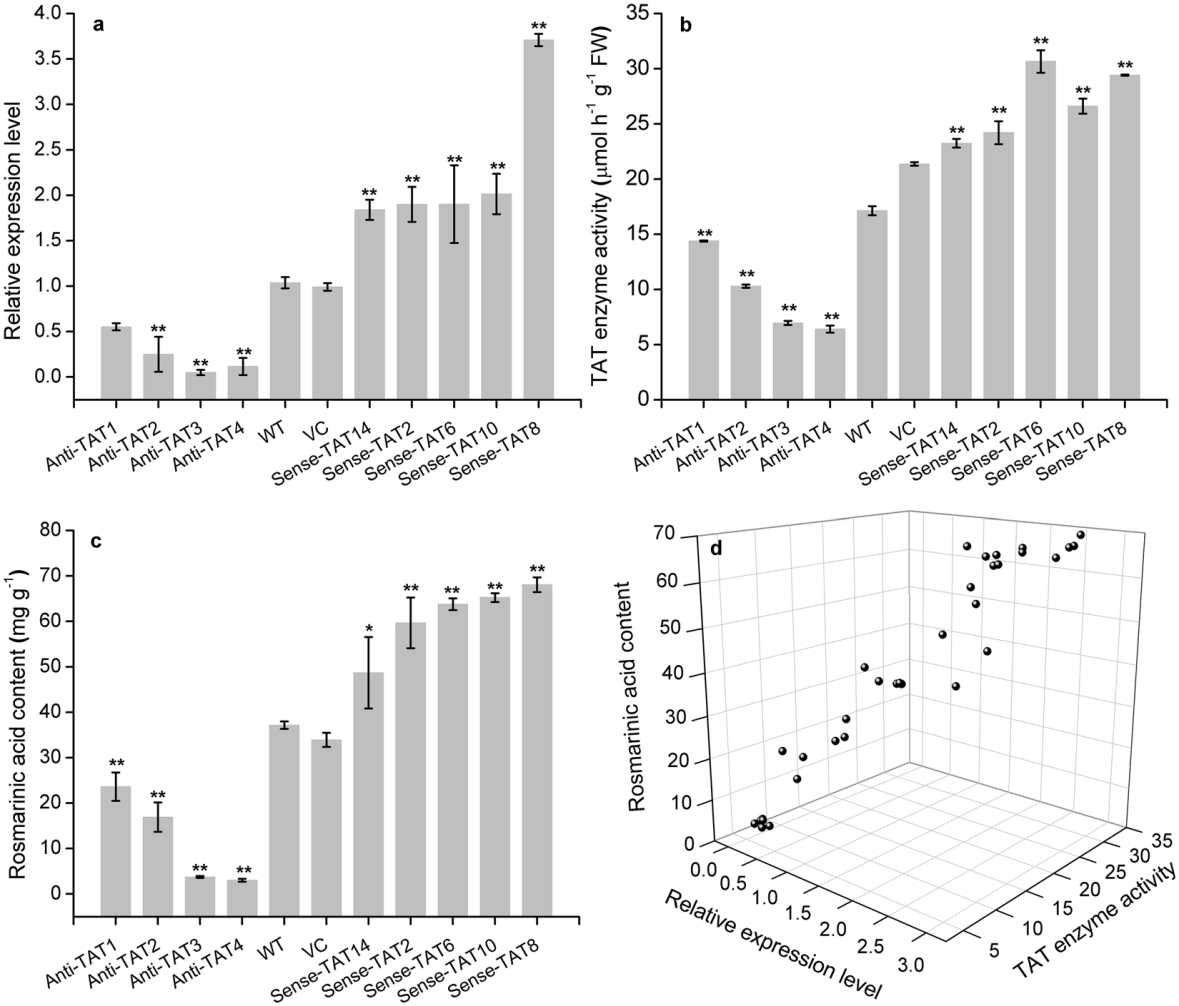
*PvTAT* gene expression level, TAT activity, RA accumulation in wild-type and transgenetic hairy root lines, and the correlation between *PvTAT* gene expression level, TAT activity, and RA accumulation in hairy root lines. (**a**) Gene expression was determined by qRT-PCR and is shown relative to the expression level of the housekeeping gene β-actin. Transcripts of *PvTAT* in the wild-type hairy root lines were set to 1. (**b**) TAT enzyme activity was measured as describled in the Method. (**c**) RA accumulation was determined by HPLC. (**d**) Shows the correlation between *PvTAT* gene expression level, TAT activity, and RA accumulation in hairy root lines. Data are means ± SD of three replicates. *P < 0.05 and **P < 0.01 as determined by Tukey HSD test. WT, the untransformed hairy roots lines (wild-type control); VC, empty vector transformed hairy roots lines (vector control); Anti-TAT, antisense-expressed TAT hairy root lines; Sense-TAT, sense-expressed TAT hairy root lines.

To determine whether PvTAT is involved in RA biosynthesis *in planta*, we used an antisense/sense expression-based approach to develop transgenic hairy root lines with regulated levels of *PvTAT* transcription. Wild-type hairy root lines with untransformed *A*. *rhizogenes* ATCC 15834 and hairy root lines transformed with empty vector pCAMBIA2300 were used as wide-type control and vector control, respectively. PCR amplification of *rol*B*/*C gene, *nptII*, and *PvTAT* confirmed the transformed lines (Supplemental Fig. S5). Despite the relatively low transgenic rate, four antisense and five sense *PvTAT*-expressing hairy root lines were developed that showed 46–95% decreases and 0.77–2.6-fold increases, respectively, in transcription levels relative to the wild-type lines (Fig. 4a). To investigate the degree of decrease/increase in total TAT activity due to the antisense/sense expression of *PvTAT*, we assayed TAT activity in the hairy root lines. Total activity was reduced by 23–67% in antisense-expressing hairy root lines and increased by 43–60% in sense-expressing hairy root lines (Fig. 4b), consistent with the expectation that the antisense/sense expression of *PvTAT* leads to loss/gain of TAT activity. Reduced *PvTAT* expression and activity resulted in a decrease in RA levels by 36–92% in all downregulated lines. Increased *PvTAT* expression and activity led to an increase in RA accumulation by 31–83% in all overexpressing lines (Fig. 4c). The positive relationship between *PvTAT* expression, TAT activity, and RA accumulation suggests that PvTAT serves an important role in the production of RA in *P*. *vulgaris* (Fig. 4d). Together, these results provide *in vivo* evidence of the major role of PvTAT in RA biosynthesis in *P*. *vulgaris*.

## Discussion

In plants, many substances are derived from tyrosine, such as RA, homogentisate, tocopherol, and BIA, which contribute to human health. RA and its numerous derivatives have many potential nutraceutical and pharmaceutical applications^11^. Since RA was first discovered and isolated in *Rosmarinus officinasis* ^59^, researchers have been studying its biogenesis. Because of its biological and pharmaceutical importance, RA has been studied in many medicinal plant species. TAT genes involved in RA biosynthesis have been cloned from *C*. *blumei*, *S*. *miltiorrhiza* ^15^, and *P*. *frutescens* ^53^. Aminotransferase activity are also implicated in the biosynthesis of aromatic volatiles, tocopherol, BIA, and tropane alkaloid and have been characterised in melons, Arabidopsis, *P*. *somniferum*, and *A*. *belladonna* ^34, 42–44, 46, 50, 51^, respectively. Here, we cloned the entry-point enzyme TAT from *P*. *vulgaris*, a member of the Labiatae. Through the isolation and characterisation of TAT from *P*. *vulgaris*, we provide biochemical and physiological evidence for the involvement of 4-HPP as an intermediate in RA biosynthesis (Fig. 1).

Based on a phylogenetic tree of sequence similarity, plant, mammalian, and bacterial TATs all differ from each other despite being grouped in the same subdivision within the same subgroup of the class I aminotransferases^60, 61^. Conserved amino acid residues responsible for catalysis and binding PLP are found in all proteins (Fig. 2a). Among them, Lys residue is the catalytic sites interacting with the pyridine nitrogen of PLP. Lys-246 may perform this catalytic function in PvTAT. Arg in the highly conserved attachment site is one of the residues responsible for the interaction of Tyr with TAT^40^, and Arg-254 may play this role in PvTAT (Fig. 2a). Although PvTAT shares only 15% amino acid identity with *E*. *coli* TAT, it was able to complement the *E*. *coli* mutant DL39^58^ (Fig. 3a). This was also the case for Arabidopsis At5g36160, At5g53970, and PhPPY-AT^33, 42, 45^. None these are involved in the anabolic pathways for leucine, valine, isoleucine, and aspartate (Fig. 3c).

In plants, despite L-Phe and L-Tyr are synthesised in plasmids from prephenate, which is derived from chorismate formed through the shikimate pathway^62^. Secondary metabolites, like phenolic acid, flavonoids, volatiles, alkaloids, tocopherols, and cyanogenic glycoside and proteins are derived from the aromatic amino acids L-Phe and L-Tyr^63^. However, plants have retained multiple TATs that likely utilise L-Phe and L-Tyr as substrates and are not plastid localised, but rather cytosol localised. A petunia PhPPY-AT localised in the cytosol was able to directly catalyse the formation of L-Phe from phenylpyruvate using L-Tyr as the amino donor, yielding 4-HPP as a by-product^45^. Moreover, an *A*. *belladonna* ArAT4, which is not localised to any organelle, was able to directly catalyse the formation of L-Tyr from 4-HPP using L-Phe as the amino donor, yielding phenylpyruvate as a by-product^50^. The two reactions are mutually inverse and are analogous to the formation of L-Phe and L-Tyr in bacteria, demonstrating the existence of a second, direct, extraplastidic route to L-Phe and L-Tyr biosynthesis in plants and leading to a proposed role for PhPPY-AT and Ab-ArAT4 in modulating aromatic amino acid homeostasis^45, 50^. Arabidopsis TAT genes (At2g24850, At5g53970) were both shown to encode the enzyme capable of interconverting L-Tyr and 4-HPP, as well as L-Phe and phenylpyruvate, catalysing the first step in tocopherol biosynthesis^45, 64^. Another Arabidopsis TAT gene (At5g36160) was also reported to be capable of interconverting L-Tyr and 4-HPP, as well as L-Phe and phenylpyruvate^46^. More recently, Wang, *et al*.^48^ reported that Arabidopsis At5g53970 favoured the direction of Tyr deamination to 4-HPP but At5g36160 preferred the reverse reaction, both were localised outside of the plastids. The opium poppy TAT was as an aminotransferase capable of interconverting L-Tyr and 4-HPP, as well as L-Phe and phenylpyruvate, and contributed to BIA biosynthesis^34^. Additionally, an *E*. *sinica* aromatic aminotransferases (EsAroAT1) was showed to function as opium poppy TAT, but is unlikely to contribute to ephedrine alkaloid biosynthesis through the catabolism of L-Phe to phenylpyruvate as a precursor to benzaldehyde^51^. Herein, PvTAT was also confirmed to localise in the cytoplasm and is an aminotransferase capable of interconverting L-Tyr and 4-HPP, interconverting L-Phe and phenylpyruvate (Fig. 2e, Supplemental Fig. S3, Table 1). Antisense expression of PvTAT led to dramatically decreased RA accumulation (Fig. 4c), confirming the involvement of this enzyme in the formation of RA in *P*. *vulgaris*.

Although we cannot role out that PvTAT may have amino donor substrates other than L-Tyr, L-Phe, and L-Trp, we demonstrated its strong preference for L-Tyr over the structurally related amino acid L-Phe and L-Trp (Table 1), similar behavior with *A*. *officinalis* TATs^14^, Arabidopsis At5g36160 and At5g53970^45, 46, 48^, opium poppy TAT^34^, and *E*. *sinica* AroAT1^51^. A recombinant TAT from *T*. *cruzi* was shown to have both tyrosine aminotransferase and alanine aminotransferase activities, displaying an extended substrate enzyme activity^65^. Traditionally, TAT enzymes have been characterised with aliphatic keto acid (α-ketoglutarate, oxaloacetate, and pyruvate) as acceptors and α-ketoglutarate or oxaloacetate was considered as the preferred keto acid acceptor^14, 33, 34, 46^. However, this perception has been changed since the characterisation of PhPPY-AT and CmArAT1 showed a preference for phenylpyruvate rather than α-ketoglutarate, and interconnected Tyr and Phe metabolism in petunia and melon^45^, more and more aminotransferases were found with similar behavior. Ab-ArAT4 has been characterised to prefer the conversion of 4-HPP to Tyr using Phe amino donor, also interconnecting Tyr and Phe metabolism in *A*. *belladonna* ^50^. What’s more, Arabidopsis At5g53970 was showed phenylpyruvate preference and may contribute to the cytosolic formation of Phe, like for phenylpropanoid biosynthesis, similar to its homolog in petunia^48^. Our work shows that PvTAT shows a substrate preference toward Tyr and phenylpyruvate (Table 1). However, the *K*_m_ (approx. 4 mM) for the best acceptor substrate (phenylpyruvate) is high and there may be more preferred substrates *in vivo*. Based on the presented data, PvTAT appears to be not an enzyme with high efficiency when compared with Arabidopsis At5g53970 and Ab-ArAT4 but possesses similar catalytic efficiencies to Arabidopsis At5g36160 and PhPPY-AT.

Many attempts have been made to engineer increased RA production by diverting more primary metabolites toward the production of 4-hydroxyphenylpyruvate or by the regulation of biotic/abiotic factors. Co-expression of *SmTAT and SmHPPR* in *S*. *miltiorrhiza* increased RA by 4.3-fold, although overexpression of *SmTAT* alone did not increase RA content^52^. Overexpression of the *PfTAT* gene from *P*. *frutescens* led to RA levels to be 1.5–2.0 folds higher than controls^53^. By combining the genetic manipulation of the ectopic expression of AtPAP1 and co-suppression of the cinnamoyl-CoA reductase and caffeic acid O-methyltransferase genes in *S*. *miltiorrhiza*, a 2.49-fold increase of RA was obtained^66^. Our data showed that overexpression of the *PvTAT* in hairy roots increased *TAT* expression, followed by increased enzyme activity, and and 31–83% increases in RA accumulation (Fig. 4c). In contrast, antisense expression of *PvTAT* successfully suppressed *TAT* expression, followed by decreased enzyme activity and RA accumulation, especially in antisense line 3 and 4, with 90% and 92% decreases in RA accumulation (Fig. 4c). The positive relationship between *PvTAT* mRNA levels, total TAT activity, and RA accumulation suggest that PvTAT has an important role in the production of this phenolic acid in *P*. *vulgaris*. The biochemical characterisation of a recombinant TAT *in vitro*, coupled with the physiological evaluation of function *in planta*, supports the role of 4-HPP as an intermediate in the formation of RA precursors.

Together, we presented the isolation and characterisation a tyrosine aminotransferases cDNA (*PvTAT*) from *P. vulgaris*, commonly known as “heal-all”. We have provided comprehensive information for PvTAT expression and test for the catalytic activities *in vitro* and *in vivo*, which fill in the gap in PA-producing plants. What’s more, the physiological evidence that PvTAT important for the synthesis of rosmarinic acid (RA) in *P*. *vulgaris* was confirmed by modest correlation between transcript level, TAT activity, and RA accumulation. These results highlight the RA biosynthesis pathway in *P*. *vulgaris*, and provide useful information to engineer natural products. These observations open an opportunity to modulate the level of this biotechnologically and pharmaceutically important phenolic acid through the modulation of *PvTAT* expression.

## Materials and Methods

### *P*. *vulgaris* RNA and DNA isolation and cDNA synthesis

Total RNA from *P*. *vulgaris* leaves was isolated using the RNAprep pure Plant Kit (TIANGEN, Beijing, China) according to the manufacturer’s protocol and reverse transcribed to generate cDNA using the PrimeScript RT reagent kit (Takara, Japan). Genomic DNA was isolated from *P*. *vulgaris* leaves using a DNA isolation kit (Omega, USA) following the manufacturer’s protocol.

### Amplification of PvTAT cDNA

Homologous cloning was used to obtain the ORF sequence of *P*. *vulgaris*. The same Labiatae family members of *S*. *miltiorrhiza* (KM575934.1) and *C*. *blumei* (AJ458993.1) *TAT* sequences were used as the query sequence to locally blast against *P*. *vulgaris* transcriptome database (Genebank SRX130243) with BioEdit (v7.0.9). Unigenes with more than 80% similarity were chose and assembled using SeqMan of DNAStar. A joint and predicted *PvTAT* ORF sequence was then obtained. A PCR reaction was carried out using the forward primer (5′-ATGGAGTTGCAGAGCTCGGCGCAG-3′) and the reverse primer (5′-TTAGTAGGAGTGTCGTTCGCAGAAC-3′) to amplify *PvTAT* ORF sequence. Using the same primer pairs, the DNA sequence was amplified using the DAN as a template.

### Multiple sequence alignment and phylogenetic analysis

Amino acid alignments were performed using DNAMAN 7.0. An unrooted neighbour-joining phylogenetic tree was constructed using MEGA version 5.05 with the Poisson model and default parameters. A bootstrap test of 1000 replicates was used to value the reliability of the phylogeny. The proteins used for sequence alignments and phylogenetic tree construction are available in Supplemental Table S1.

### Transcription analysis of *PvTAT*

Plant materials were described in Ru, *et al*.^67^. At the reproductive stage, leaves, stems, roots, and spikes were separately sampled and kept at −80 °C before use. Extraction and reverse-transcription of total RNA were carried out as mentioned above. Gene expression was determined by qRT-PCR following our previous method^68^ and is shown relative to the expression level of the housekeeping gene β-actin. Relative expression was calculated using the 2^−ΔΔt^ method. Transcript levels of related genes in the spike were set to 1. Data are means ± SD of three replicates of three independent individuals. Tukey HSD test was used to compare the difference among tissues. Primers were listed in Supplementary Table S2.

### Subcelluar localisation analysis of PvTAT

The full-length ORF sequence introduced *Xho*I and *Nco*I restriction sites was subcloned into the PC0390-pA7-GFP plasmid to produce TAT-GFP fusion proteins. Construction of recombinant plasmid and transient transformation of tobacco were referenced to our previous method^67^. Tobacco protoplasts and leaves were both observed for the determination of PvTAT locolisation. Images were acquired using a confocal microscope (Nikon, Japan) and software of NIS-Elements Viewer 4.0. The PC0390-pA7-GFP plasmid was used as a marker. GFP was excited at 488 nm and collected over a 500–550 nm bandpass. Chlorophyll fluorescence was excited at 638 nm and collected over a 662–737 nm bandpass.

### Expression and purification of recombinant proteins

The pET32a-TAT plasmid encoding PvTAT was constructed by PCR amplification of the *PvTAT* ORF sequence that introduced *Eco*RI and *Hin*dIII restriction sites. The PCR fragments were subcloned into the PMD19T Simple cloning vector (Takara) and checked by sequencing. The PMD19T-TAT plasmid and the pET32a (NEB, England) vector were digested by *Eco*RI-*Hin*dIII restriction enzymes to produce the fusion protein with an N-terminal His tag. The resulting plasmid was termed pET32a-TAT and transformed into the *E*. *coli* strain Rosetta (DE3).

*Escherichia coli* strain Rosetta (DE3) cells carrying the pET32a-TAT plasmids or the pET32a empty plasmids were grown at 37 °C in 500 ml of Terrific-Broth supplemented with 34 mg l^−1^ chloramphenicol and 100 mg l^−1^ carbenicillin. When the culture reached an OD_600_ of ~0.6, isopropyl-β-1-D-thiogalactoside (1 mM) was added to a final concentration of 0.3 mM to induce protein expression and the culture was incubated for 6 h. The cells were harvested by centrifugation at 10,000 g for 10 min at 4 °C. The pellet was washed twice with PBS buffer, suspended in 200 mM Tris, 100 mM KCl, and 10% glycerol (w/v), pH 8.0, and lysed by high-pressure broken using a cell disrupter (JN-02C, JNBIO, Guangzhou, China). The crude extract was centrifuged at 12,000 g for 20 min at 4 °C to yield a cell-free supernatant. The supernatant was used for affinity purification over His-Tag purification resin (Solaibao, Beijing, China). Proteins eluted with 250 mM imidazole were analysed by SDS-PAGE on a 12% (w/v) acrylamide gel and visualised using Coomassie Brilliant Blue R-250 staining. Protein concentrations were determined using a Bradford Protein Assay Kit (Takara).

Western blotting analysis was determined as Wang, *et al*.^69^ described previously, except the protein was transferred to NC membrane (Solarbio). The membrane was incubated with anti-His antibodies at a dilution of 1:3000 in blocking buffer for 1 h, after washing with TBST buffer for 10 min, the membrane was then incubated with goat anti-rabbit IgG alkaline phosphatase-conjugated as secondary antibodies (1:3000 dilution) for 1 h. Antigen-antibody complex was visualised using the BCIP/NBT kit (Beyotime, China).

### Enzyme assay

To measure TAT activity in *P*. *vulgaris* hairy root lines, proteins were extracted and determined as Lopukhina, *et al*.^42^ described previously with minor modification. Hairy roots (0.2 g fresh weight) were extracted with 3 ml of ice-cold buffer, containing 100 mM potassium phosphate buffer, 0.1 mM EDTA, 8.0 mM α-ketoglutarate, 0.2 mM pyridoxal-5-phosphate (PLP), and 1 mM dithiothreitol, pH 8.0. After centrifugation at 12,000 g at 4 °C for 10 min, the supernatant was incubated on ice and used as a crude extract enzyme. The reaction mixture containing 125 mM KH_2_PO_4_/K_2_HPO_4_ buffer, 5.5 mM L-Tyr, 0.75 mM EDTA, 0.1 mM PLP, and 10 mM α-ketoglutarate, pH 8.0, was pre-incubated at 30 °C for 30 min. The reaction was initiated by adding 200 μl of crude protein and incubated at 30 °C in a total volume of 3 ml. After 30 min the reaction was terminated with 1 ml of 10 M KOH and extinction of 4-hydroxybenzaldehyde (product derivative) was measured exactly 30 min later at 331 nm using an ultraviolet-visible spectrophotometer (UV-1700, SHIMADZU, Japan). The extinction coefficient used for 4-hydroxybenzaldehyde was 24 900 L mol^−1^cm^−1^ ^33^.

To estimate the pH optimum for the recombinant protein, pH values were varied between 6 and 10 and 5 μl (0.2 μg) of purified protein was assayed as described above in a total volume of 1 ml. Enzyme kinetics were obtained by monitoring the absorbance of various transamination reaction products 4-HPP at 331 nm, phenylpyruvate at 320 nm, and indole-3-pyruvate at 328 nm corresponding to the substrates L-Tyr, L-Phe, and L-Trp. The assay contained 125 mM KH_2_PO_4_/K_2_HPO_4_ buffer, 0.75 mM EDTA, 0.1 mM PLP, and various substrate concentrations, pH 8.0, and incubated at 30 °C in a total volume of 1 ml. When α-ketoglutarate (10 mM) was the amino acceptor, 0–4 mM L-Tyr, 0–50 mM L-Phe, and 0–50 mM L-Trp were used as the amino donor. When L-Tyr (5.5 mM) was the amino donor, 0–100 mM α-ketoglutarate, 0–200 mM pyruvate, 0–300 mM oxaloacetate, and 0–40 mM phenylpyruvate were used as the amino acceptor.

For the reverse reaction, the assay was determined as Prabhu and Hudson^46^ described. The 100 μl assay mixture consisted of 125 mM KH_2_PO_4_/K_2_HPO_4_ buffer, 0.3 mM NAD, 0.3 mM CoA, 10 mM glutamate, 0.75 mM EDTA, 100 μg 2-ketoglutarate dehydrogenase (0.6 U mg^*−*1^ protein) (Sigma), 0.02 μg pure recombinant protein, pH 8.0. After incubated at 30 °C for 30 min, 0–4 mM phenylpyruvate, 0–10 mM 4-hydroxyphenylpyruvate, or 0–40 mM oxaloacetate was added to initiate the reaction. NADH formation was measured at 340 nm using a Multi-Mode Microplate Readers (Spectra Max M 2, Molecular Devices, USA). The extinction coefficient used for NADH was 6220 L mol^−1^cm^−1^ ^33^.

*V*_max_ and *K*_m_ values were calculated according to a nonlinear regression of the Michaelis-Menten equation, where *V* = (*V*_max_S)/(*K*_m_ + S)^70^. For the calculation of *k*_cat_, a molecular weight of 65 kDa was assumed for the His-tagged TAT protein. All enzyme assays were performed at an appropriate enzyme concentration so that reaction velocity was linear and proportional to enzyme concentration during the incubation time period. Kinetic data were evaluated by curveExpert 1.4. At least triplicate assays were performed for all data points.

### Functional complementation of the *E*. *coli* DL39 mutant

The pET32a-TAT and pBAD33^57^ were both digested by *Xba*I-*Hin*dIII restriction enzymes to produce the pBAD33-TAT constructs. The *E*. *coli* auxotrophic mutant DL39 (no. 6913)^58^ with genotype (LAM-, aspC13, fnr-25, rph-1, ilvE12, tyrB507), obtained from the Coli Genome Stock Center (CGSC) (http://cgsc.biology.yale.edu/), was used for the complementation assays and transformed with either the pBAD33 or pBAD33-TAT constructs. Procedure of complementation assays were performed essentially as Prabhu and Hudson^46^ previously described.

### Antisense and sense expression of *PvTAT in planta*

To construct pCAMBIA2300-TAT sense and antisense expression vectors, the *PvTAT* ORF, introduced *Xba*I and *Kpn*I restriction sites, was inserted into the pCAMBIA2300 vector in sense and antisense orientations. Then, the pCAMBIA2300-TAT and pCAMBIA2300 plasmids were transformed into an *A*. *rhizogenes* strain ATCC 15834 using the heat shock method. Positive clones were confirmed by PCR and restriction enzymes digestion. Plant transformation was carried out according to our previous method^68^, except that the transformants were screened with cefotaxim solution and kanamycin together.

### Identification of transgenic hairy roots by PCR

DNA was isolated from wild-type and transgenic hairy roots using a genomic DNA isolation kit (Omega) according to the manufacturer’s protocol. Four primer pairs, *rol*B/C, *nptII*, 35S forward primer 1(35S F1), and STAT reverse primer (STAT R) were designed for the identification of sense-expressing hairy root lines. 35SF was located at the 35S promoter of the pCAMBIA2300 vector and STATR was from the *PvTAT* gene. 35S forward primer 2 (35SF2) and ATAT reverse primer (ATATR) were designed for the identification of antisense-expressing hairy root lines. ATATR was also from the *PvTAT* gene. Primers were shown in Supplemental Table S2.

### HPLC assay for RA content

RA accumulation in wild-type hairy roots and transgenetic hairy root lines was assayed using HPLC according to our previous method^68^.

### Analysis for *PvTAT* gene expression

qRT-PCR analysis for *PvTAT* gene expression in wild-type hairy roots and transgenetic hairy root lines was determined as mentioned above. Transcripts of *PvTAT* in the wild-type hairy root lines were set to 1.

### Data analysis

One-way analysis of variance (ANOVA) was performed to highlight differences in wide-type hairy root lines, hairy root lines transformed with empty vector, and transgenic hairy root lines followed by ‘Tukey HSD’ post-hoc multiple comparison tests. Normality and homogeneity of variance of data were assessed prior to analysis. All the data analyses were accomplished using the “Statistical Package” for Social Sciences program (SPSS 16.0, SPSS Inc., USA). OriginPro8.0 (OriginLab Corporation, Northampton, MA) was used for graphical presentation. All the data were expressed as means ± SD with triplicate replicates.

## Electronic supplementary material

Supplementary information

## References

[1] Psotová J, Kolář M, Soušek J, Švagera Z (2003). Biological activities of Prunella vulgaris extract. Phytotherapy Research.

[2] Jeong S, Park H, Hong S, Yum S (2015). Lipophilic modification enhances anti-colitic properties of rosmarinic acid by potentiating its HIF-prolyl hydroxylases inhibitory activity. European Journal of Pharmacology.

[3] Erkan N, Ayranci G, Ayranci E (2008). Antioxidant activities of rosemary (*Rosmarinus Officinalis* L.) extract, blackseed (Nigella sativa L.) essential oil, carnosic acid, rosmarinic acid and sesamol. Food Chem.

[4] Bakota EL, Winkler-Moser JK, Berhow MA, Eller FJ (2015). Antioxidant Activity and Sensory Evaluation of a Rosmarinic Acid-Enriched Extract of *Salvia officinalis*. J Food Sci.

[5] Tsai TH, Chuang LT, Lien TJ, Liing YR (2013). Rosmarinus officinalis Extract Suppresses Propionibacterium acnes-Induced Inflammatory Responses. Journal of Medicinal Food.

[6] Kuruuzum-Uz A, Suleyman H, Cadirci E, Guvenalp Z, Demirezer LO (2012). Investigation on anti-inflammatory and antiulcer activities of *Anchusa azurea* extracts and their major constituent rosmarinic acid. Zeitschrift fur Naturforschung. C: A Journal of Biosciences.

[7] Heo S-K, Noh E-K, Yoon D-J, Jo J-C (2015). Rosmarinic acid potentiates ATRA-induced macrophage differentiation in acute promyelocytic leukemia NB4 cells. European Journal of Pharmacology.

[8] Yesil-Celiktas O, Sevimli C, Bedir E, Vardar-Sukan F (2010). Inhibitory Effects of Rosemary Extracts, Carnosic Acid and Rosmarinic Acid on the Growth of Various Human Cancer Cell Lines. Plant food hum nutr.

[9] Tai J, Cheung S, Wu M, Hasman D (2012). Antiproliferation effect of Rosemary (*Rosmarinus officinalis*) on human ovarian cancer cells *in vitro*. Phytomedicine.

[10] Pereira P, Tysca D, Oliveira P, da Silva Brum LF (2005). Neurobehavioral and genotoxic aspects of rosmarinic acid. pharmacol Res.

[11] Kim G-D, Park YS, Jin Y-H, Park C-S (2015). Production and applications of rosmarinic acid and structurally related compounds. Applied Microbiology and Biotechnology.

[12] Bauer N, Leljak-Levanic D, Jelaska S (2004). Rosmarinic acid synthesis in transformed callus culture of *Coleus blumei* Benth. Zeitschrift fur Naturforschung. C: A J Biosci.

[13] Petersen M, Häusler E, Meinhard J, Karwatzki B (1994). The biosynthesis of rosmarinic acid in suspension cultures of *Coleus blumei*. Plant cell tiss org.

[14] De-Eknamkul W, Ellis BE (1987). Purification and characterization of tyrosine aminotransferase activities from *Anchusa officinalis* cell cultures. Archives of biochemistry and biophysics.

[15] Huang B, Yi B, Duan Y, Sun L (2008). Characterization and expression profiling of tyrosine aminotransferase gene from *Salvia miltiorrhiza* (Dan-shen) in rosmarinic acid biosynthesis pathway. Mol Biol Rep.

[16] Chen H, Chen F, Zhang Y-L, Song J-Y (1999). Production of rosmarinic acid and lithospermic acid B in Ti transformed *Salvia miltiorrhiza* cell suspension cultures. Process Biochem.

[17] Grzegorczyk I, Matkowski A, Wysokińska H (2007). Antioxidant activity of extracts from *in vitro* cultures of Salvia officinalis L. Food Chemistry.

[18] Lee SY, Xu H, Kim YK, Park SU (2007). Rosmarinic acid production in hairy root cultures of Agastache rugosa Kuntze. World Journal of Microbiology and Biotechnology.

[19] Tada H, Murakami Y, Omoto T, Shimomura K (1996). Rosmarinic acid and related phenolics in hairy root cultures of *Ocimum basilicum*. Phytochemistry.

[20] Strazzer P, Guzzo F, Levi M (2011). Correlated accumulation of anthocyanins and rosmarinic acid in mechanically stressed red cell suspensions of basil (*Ocimum basilicum*). J Plant physiol.

[21] Li W, Koike K, Asada Y, Yoshikawa T (2005). Rosmarinic acid production by *Coleus forskohliihairy* root cultures. Plant cell tiss org.

[22] Jiang J, Bi H, Zhuang Y, Liu S (2016). Engineered synthesis of rosmarinic acid in *Escherichia coli* resulting production of a new intermediate, caffeoyl-phenyllactate. Biotechnol Lett.

[23] Bloch SE, Schmidt-Dannert C (2014). Construction of a Chimeric Biosynthetic Pathway for the De Novo Biosynthesis of Rosmarinic Acid in *Escherichia coli*. ChemBioChem.

[24] Zhuang Y, Jiang J, Bi H, Yin H (2016). Synthesis of rosmarinic acid analogues in Escherichia coli. Biotechnol Lett.

[25] Ellis BE, Towers GHN (1970). Biogenesis of Rosmarinic Acid in *Mentha*. Biochem J.

[26] Razzaque A, Ellis BE (1977). Rosmarinic acid production in Coleus cell cultures. Planta.

[27] Ellis BE, Remmen S, Goeree G (1979). Interactions between parallel pathways during biosynthesis of rosmarinic acid in cell suspension cultures of Coleus blumei. Planta.

[28] Wanchai D-E, Brian EE (1987). Tyrosine aminotransferase: The entrypoint enzyme of the tyrosine-derived pathway in rosmarinic acid biosynthesis. Phytochemistry.

[29] Petersen M, Häusler E, Karwatzki B, Meinhard J (1993). Proposed biosynthetic pathway for rosmarinic acid in cell cultures of *Coleus blumei* Benth. Planta.

[30] Petersen M (2013). Rosmarinic acid: new aspects. Phytochem rev.

[31] Soll J (1987). α-Tocopherol and Plastoquinone Synthesis in Chloroplast Membranes. Methods In Enzymology.

[32] Loffelhardt W, Kindl H (1979). Conversion of 4-hydroxyphenylpyruvic acid into homogentisic acid at the thylakoid membrane of *Lemna gibba*. Febs Lett.

[33] Riewe D, Koohi M, Lisec J, Pfeiffer M (2012). A tyrosine aminotransferase involved in tocopherol synthesis in Arabidopsis. Plant J.

[34] Lee E-J, Facchini PJ (2011). Tyrosine aminotransferase contributes to benzylisoquinoline alkaloid biosynthesis in opium poppy. Plant physiology.

[35] National Pharmacopoeia Committee. *Chinese Pharmacopoeia* (Chemical Industry Press, 2015).

[36] Kim, Y. B., Shin, Y., Tuan, P. A. & Li, X. *et al*. Molecular cloning and characterization of genes involved in rosmarinic acid biosynthesis from Prunella vulgaris. *Biological and Pharmaceutical Bulletin* (2014). 10.1248/bpb.b14-00139 24739190

[37] Petersen M (1997). Cytochrome P450-dependent hydroxylation in the biosynthesis of rosmarinic acid in Coleus. Phytochemistry.

[38] Ko T-P, Wu S-P, Yang W-Z, Tsai H (1999). Crystallization and preliminary crystallographic analysis of the Escherichia coli tyrosine aminotransferase. Acta Crystallographica Section D.

[39] Blankenfeldt W, Nowick iC, Montemartini-Kalisz M, Kalisz HM (1999). Crystal structure of *Trypanosoma cruzi* tyrosine aminotransferase: substrate specificity is influenced by cofactor binding mode. Protein Science.

[40] Mehere P, Han Q, Lemkul JA, Vavricka CJ (2010). Tyrosine aminotransferase: biochemical and structural properties and molecular dynamics simulations. Protein & Cell.

[41] Moreno MA, Abramov A, Abendroth J, Alonso A (2014). Structure of tyrosine aminotransferase from Leishmania infantum. Acta Crystallographica Section F.

[42] Lopukhina A, Dettenberg M, Weiler EW, Holländer-Czytko H (2001). Cloning and characterization of a coronatine-regulated tyrosine aminotransferase from Arabidopsis. Plant physiology.

[43] Holländer-Czytko H, Grabowski J, Sandorf I, Weckermann K (2005). Tocopherol content and activities of tyrosine aminotransferase and cystine lyase in *Arabidopsis* under stress conditions. J Plant Physiol.

[44] Gonda I, Bar E, Portnoy V, Lev S (2010). Branched-chain and aromatic amino acid catabolism into aroma volatiles in *Cucumis melo* L. fruit. J Exp Bot.

[45] Yoo, H., Widhalm, J. R., Qian, Y. & Maeda, H. *et al*. An alternative pathway contributes to phenylalanine biosynthesis in plants via a cytosolic tyrosine:phenylpyruvate aminotransferase. *Nat Commun* **4**, doi:10.1038/ncomms3833 (2013). 10.1038/ncomms3833 24270997

[46] Prabhu, P. R. & Hudson, A. O. Identification and partial characterization of an L-tyrosine aminotransferase (TAT) from *Arabidopsis thaliana*. *Biochemistry research international* **2010** (2010). 10.1155/2010/549572 PMC3005984 21188077

[47] Grossmann K, Hutzler J, Tresch S, Christiansen N (2012). On the mode of action of the herbicides cinmethylin and 5-benzyloxymethyl-1, 2-isoxazolines: putative inhibitors of plant tyrosine aminotransferase. Pest manag sci.

[48] Wang M, Toda K, Maeda HA (2016). Biochemical properties and subcellular localization of tyrosine aminotransferases in Arabidopsis thaliana. Phytochemistry.

[49] Hirata H, Ohnishi T, Ishida H, Tomida K (2012). Functional characterization of aromatic amino acid aminotransferase involved in 2-phenylethanol biosynthesis in isolated rose petal protoplasts. Journal of Plant Physiology.

[50] Bedewitz MA, Góngora-Castillo E, Uebler JB, Gonzales-Vigil E (2014). A Root-Expressed l-Phenylalanine:4-Hydroxyphenylpyruvate Aminotransferase Is Required for Tropane Alkaloid Biosynthesis in *Atropa belladonna*. The Plant Cell.

[51] Kilpatrick K, Pajak A, Hagel JM, Sumarah MW (2016). Characterization of aromatic aminotransferases from *Ephedra sinica* Stapf. Amino Acids.

[52] Xiao Y, Zhang L, Gao S, Saechao S (2011). The c4h, tat, hppr and hppd Genes Prompted Engineering of Rosmarinic Acid Biosynthetic Pathway in *Salvia miltiorrhiza* Hairy Root Cultures. PLoS ONE.

[53] Lu X, Hao L, Wang F, Huang C (2013). Molecular cloning and overexpression of the tyrosine aminotransferase (TAT) gene leads to increased rosmarinic acid yield in Perilla frutescens. Plant Cell, Tissue and Organ Culture (PCTOC).

[54] Almeida J, Quadrana L, Asís R, Setta N (2011). Genetic dissection of vitamin E biosynthesis in tomato. J Exp bot.

[55] Grynkiewicz G, Gadzikowska M (2008). Tropane alkaloids as medicinally useful natural products and their synthetic derivatives as new drugs. Pharmacol rep.

[56] Mikkelsen MD, Naur P, Halkier BA (2004). Arabidopsis mutants in the C–S lyase of glucosinolate biosynthesis establish a critical role for indole-3-acetaldoxime in auxin homeostasis. The Plant Journal.

[57] Guzman L-M, Belin D, Carson MJ, Beckwith J (1995). Tight regulation, modulation, and high-level expression by vectors containing the arabinose PBAD promoter. J Bacteriol.

[58] LeMaster DM, Richards FM (1988). NMR sequential assignment of *Escherichia coli* thioredoxin utilizing random fractional deuteriation. Biochemistry.

[59] Scarpati ML, Oriente G (1958). Isolamento e costituzione dell’acido rosmarinico (dal rosmarinus off.). Riserca Science.

[60] Mehta, P. K., Hale, T. I. & Christen, P. Aminotransferases: demonstration of homology and division into evolutionary subgroups. *European Journal of Biochemistry* **2** (1993). 10.1111/j.1432-1033.1993.tb17953.x 8513804

[61] Mehta PK, Hale TI, Christen P (1989). Evolutionary relationships among aminotransferases: Tyrosine aminotransferase, histidinol-phosphate aminotransferase, and aspartate aminotransferase are homologous proteins. European Journal of Biochemistry.

[62] Maeda H, Dudareva N (2012). The Shikimate Pathway and Aromatic Amino Acid Biosynthesis in Plants. Annual Review of Plant Biology.

[63] Weng J, Philippe R, Noel J (2012). The rise of chemodiversity in plants. Science.

[64] Sandorf I, Holländer-Czytko H (2002). Jasmonate is involved in the induction of tyrosine aminotransferase and tocopherol biosynthesis in *Arabidopsis thaliana*. Planta.

[65] Montemartini M, Búa J, Bontempi E, Zelada C (1995). A recombinant tyrosine aminotransferase from *Trypanosoma cruzi* has both tyrosine aminotransferase and alanine aminotransferase activities. FEMS Microbiology Letters.

[66] Zhang Y, Yan Y-P, Wu Y-C, Hua W-P (2014). Pathway engineering for phenolic acid accumulations in *Salvia miltiorrhiza* by combinational genetic manipulation. Metabolic Engineering.

[67] Ru, M., Wang, K., Bai, Z. & Peng, L. *et al*. Molecular cloning and characterisation of two enzymes involved in the rosmarinic acid biosynthesis pathway of Prunella vulgaris L. *Plant Cell Tiss Org*, 1–10, doi:10.1007/s11240-016-1117-z (2016).

[68] Ru M, An YY, Wang KR, Peng L (2016). *Prunella vulgaris* L. hairy roots: Culture, growth, and elicitation by ethephon and salicylic acid. Eng life sci.

[69] Wang X, Sun Y, Yang H, Lu Y (2016). Oxidized Low-Density Lipoprotein Induces Apoptosis in Cultured Neonatal Rat Cardiomyocytes by Modulating the TLR4/NF-κB Pathway. Sci Rep.

[70] Hernández A, Ruiz MT (1998). An EXCEL template for calculation of enzyme kinetic parameters by non-linear regression. Bioinformatics.

