# Supplementary material for: A tyrosine aminotransferase involved in rosmarinic acid biosynthesis in Prunella vulgaris L
Source: Sci Rep. 2017 Jul 7;7:4892. doi: 10.1038/s41598-017-05290-4 (PMC5501851; doi:10.1038/s41598-017-05290-4)
Supplement: Supplementary file 1 — Supplementary information [file 41598_2017_5290_MOESM1_ESM.doc]

A tyrosine aminotransferase involved in rosmarinic acid biosynthesis in *Prunella vulgaris* L.

Mei Ru^1^, Kunru Wang^1^, Zhenqing Bai^1^, Liang Peng^2^, Shaoxuan He^3^, Yong Wang^1^, Zongsuo Liang*^1,4^

^1^Institute of Soil and Water Conservation, Chinese Academy of Sciences&Ministry of Water Resources, Yangling 712100, P.R.China

^2^College of Pharmacy, Shannxi University of Chinese Medicine, Xi’an 710000, P.R.China

^3^Ecological Environmental Monitoring Station, Environmental Protection Agency, Dazu 402360, P.R.China

^4^College of Life Sciences, Zhejiang Sci-Tech University, Hangzhou 310000, P.R.China

*Corresponding author: Zongsuo Liang, Institute of Soil and Water Conservation, Chinese Academy of Sciences&Ministry of Water Resources, Yangling 712100, People’s Republic of China.

E-mail: [liangzs@ms.iswc.ac.cn](mailto:liangzs@ms.iswc.ac.cn), Tel: +86 029-87092262 Fax:+86 029-87092262

Table S1. Proteins used for multiple sequence alignment and phylogenetic analysis^a^.

^a^Phylogenetic analysis of these proteins is presented in Figure 2b.

| Species | Gene name | Identifier |
| --- | --- | --- |
| *Prunella vulgaris* | PvTAT | AJW87632 |
| *Salvia miltiorrhiza* | SmTAT | ABC60050 |
| *Solenostemon scutellaridoides* | SsTAT | CAD30341 |
| *Perilla frutescens* | PfTAT | ADO17550.1 |
| *Scutellaria baicalensis* | SbTAT | AIV98132.1 |
| *Solanum pennellii* | SpTAT | ADZ24702.1 |
| *Atropa belladonna* | AbArAT1 | AHN10101.1 |
| *Atropa belladonna* | AbArAT4 | AHN10104.1 |
| *Atropa belladonna* | AbArAT5 | AHN10105.1 |
| *Medicago truncatula* | MtTAT | AAY85183.1 |
| *Glycine max* | GmTAT | AAY21813.1 |
| *Theobroma cacao* | TcTAT | XP_007021573.1 |
| *Oryza sativa* | OsTAT | BAF95202.1 |
| *Papaver somniferum* | PsTyrAT | ADC33123.1 |
| *Populus trichocarpa* | PtTAT | XP_002328046.1 |
| *Ricinus communis* | RcTAT | XP_002517869 |
| *Arabidopsis thaliana* | AtTAT1 | AAK82963.1 |
| *Arabidopsis thaliana* | AtTAT2 | AF301899_1 |
| *Arabidopsis thaliana* | AtTAT3 | NP_180058.1 |
| *Arabidopsis thaliana* | AtTAT4 | NP_194090.2 |
| *Arabidopsis thaliana* | AtTAT5 | NP_198465.3 |
| *Arabidopsis thaliana* | AtTAT6 | NP_001031739.1 |
| *Arabidopsis thaliana* | AtTAT7 | NP_200208.1 |
| *Cucumis melo* | CmArAT1 | ADC45389 |
| *Petunia x hybrida* | PhPPY-AT | AHA62827.1 |
| *Ephedra sinica* | EsAroAT1 | AGK24944.1 |
| *Homo sapiens* | HsTAT | NP_000344.1 |
| *Mus musculus* | MmTAT | NP_666326.1 |
| *Danio rerio* | DrTAT | NP_001071022.1 |
| *Caenorhabditis elegans* | CeTAT | NP_510454.1 |
| *Drosophila melanogaster* | DmTAT | NP_572953.1 |
| *Salmonella enterica* | SeTAT | CAD09232.1 |
| *Bordetella petrii* | BpTAT | CAP43123.1 |
| *Escherichia coli* | EcTAT | gi|5822529| |
| *Pseudomonas aeruginosa* | PaTAT | AAD45270.1 |

Table S2. Primers used in this study.

| Pimers | Forward (5’→3’) | Reverse (5’→3’) | Product size (bp) |
| --- | --- | --- | --- |
| *(qRT)TAT* | CATTCATACAGGCTGCAGTTCC | CCAGTTCTTTAGACCCACAGC | 336 |
| *rol*B | GCTCTTGCAGTGCTAGATTT | GAAGGTGCAAGCTACCTCTC | 423 |
| *rol*C | CTCCTGACATCAAACTCGTC | TGCTTCGAGTTATGGGTACA | 626 |
| *nptΠ* | ATGGGGATTGAACAAGATGG | TCAGAAGAACTCGTCAAGAAG | 798 |
| *35SF1*  *STATR* | GAGGACCTAACAGAACTCGCC | CCAAAAGCCCCAAAATCCCCT | 598 |
| *35SF2*  *ATATR* | GACGCACAATCCCACTATCC | GCTGTGGGTCTAAAGAACTGG | 194 |


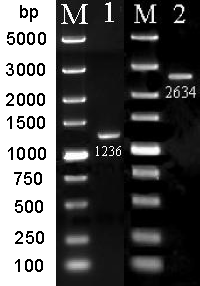


Figure S1. Gene cloning of tyrosine aminotransferase (PvTAT) from *P. vulgaris*. *Lane* 1, cDNA sequence of *PvTAT*. *Lane* 2, DNA sequence of *PvTAT*.


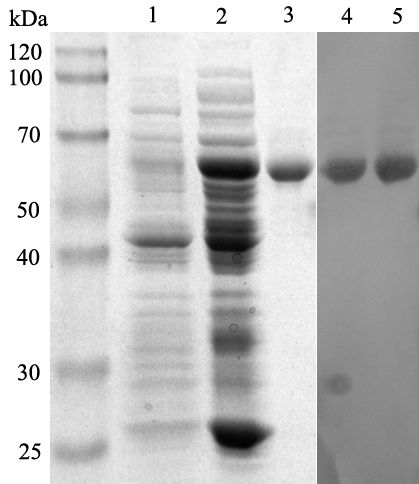


Figure S2. Purification of His-tagged, recombinant PvTAT from *E. coli* strain Rosetta (DE3). N-terminally His-tagged PvTAT (65 kDa) (*lane 3*) was purified (*lane 2*) with His-Tag puriﬁcation resin from crude extract (*lane 1*) using 250 mM imidazol solutions. Crude protein extracts from *E. coli* cells were harvested after 6 h induction with 0.3 mM isopropyl-D-thiogalactopyranoside (IPTG) and grown at 37°C. Lanes with 15 μl of each fraction were separated by SDS-PAGE on a 12% (w/v) acrylamide gel and visualised using Commassie Brilliant Blue G-250 staining. The purified protein was analysed for its intactness by western blots uses anti-His antibody (1:3000 dilution) that is shown in *lane 4 and 5*.


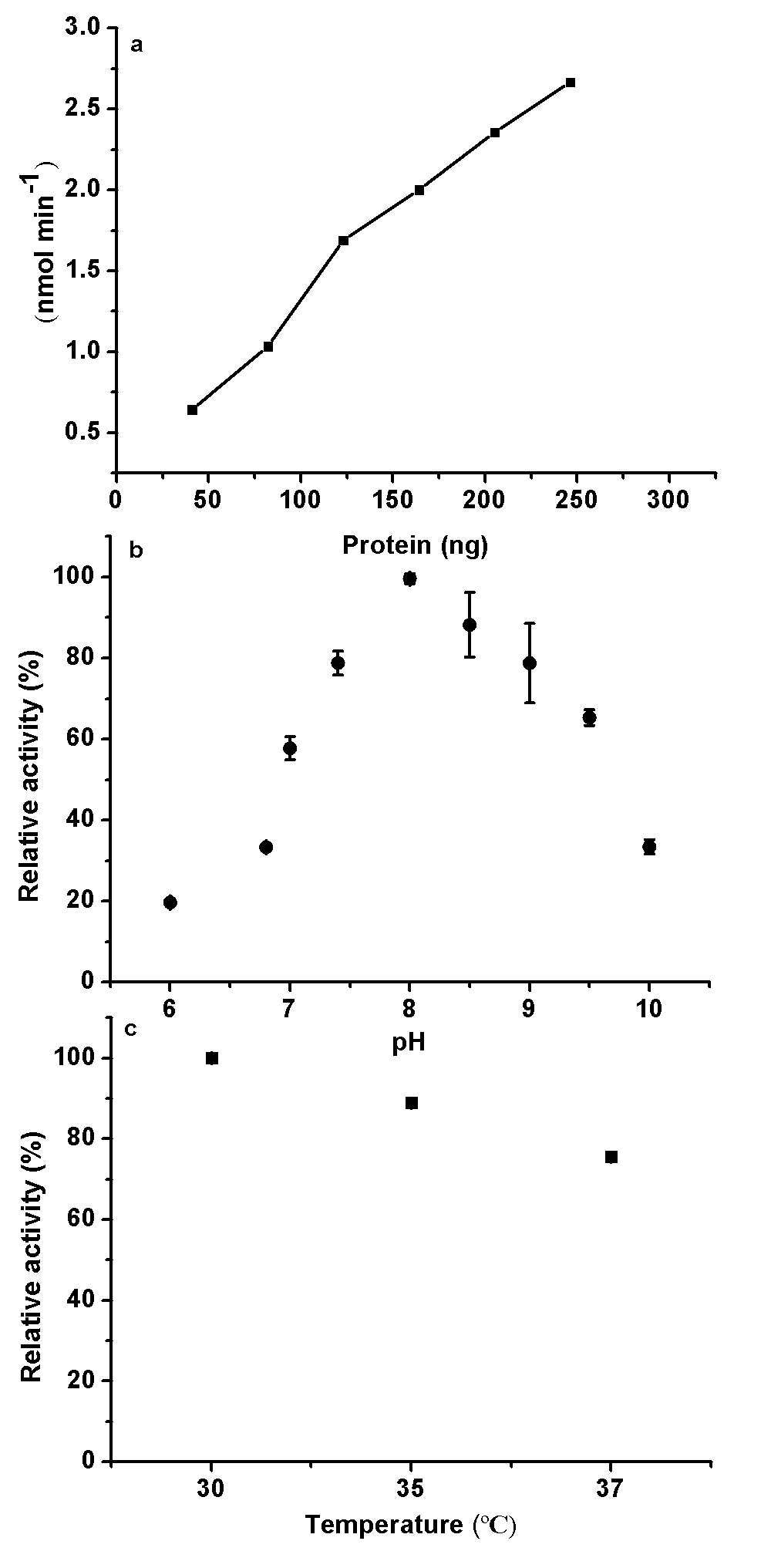


Figure S3. Detection of recombinant PvTAT activity (a) and the effect of pH (b) and temperature (c) on its activity. To estimate the temperature optimum for the recombinant protein, 30 °C, 35°C, and 37°C were varied. 30 °C and pH 8.0 were used to measure protein activity. The reaction mixture containing 125 mM KH_2_PO_4_/ K_2_HPO_4_ buffer, 5.5 mM L-Tyr, 0.75 mM EDTA, 0.1 mM pyridoxal-phosphate and 10 mM α-ketoglutarate, were pre-incubated at 30°C for 30 min. The reaction was initiated by adding 5 μl (0.2 μg) protein and incubated at 30°C in a total volume of 1 ml. After 30 min the reaction was terminated with 100 μl of 10 M KOH and extinction of 4-hydroxybenzaldehyde (product derivative) was measured exactly 30 min later at 331 nm using an [ultraviolet](app:ds:ultraviolet) visible [spectrophotometer](app:ds:spectrophotometer) (UV-1700, SHIMADZU, Japan). The extinction coefficient used for 4-hydroxybenzaldehyde was 24900 L mol^-1^cm^-1^ [^1^](#_ENREF_1).

1 Riewe, D., Koohi, M., Lisec, J., Pfeiffer, M. et al. A tyrosine aminotransferase involved in tocopherol synthesis in Arabidopsis. *PLANT J* **71**, 850-859 (2012).


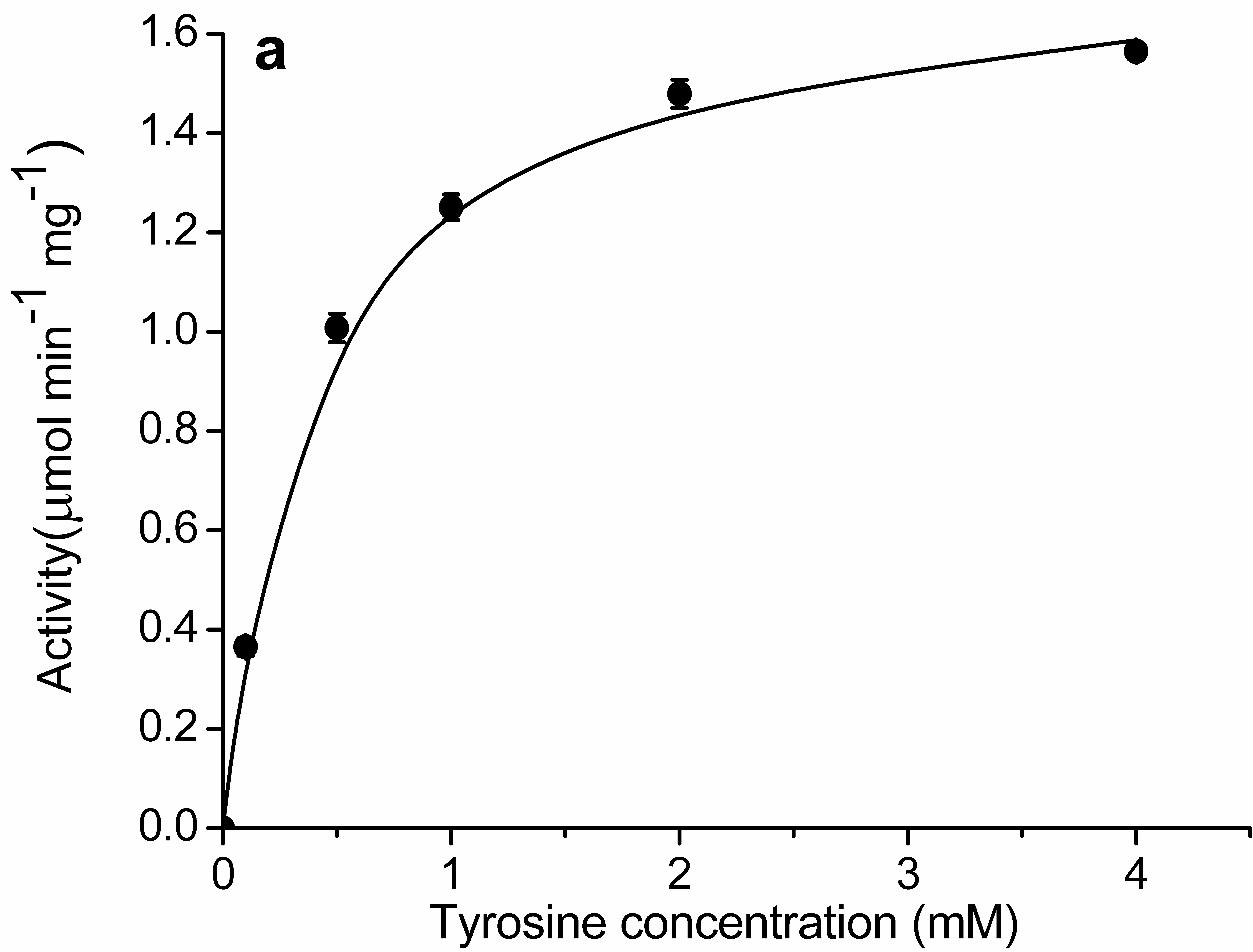

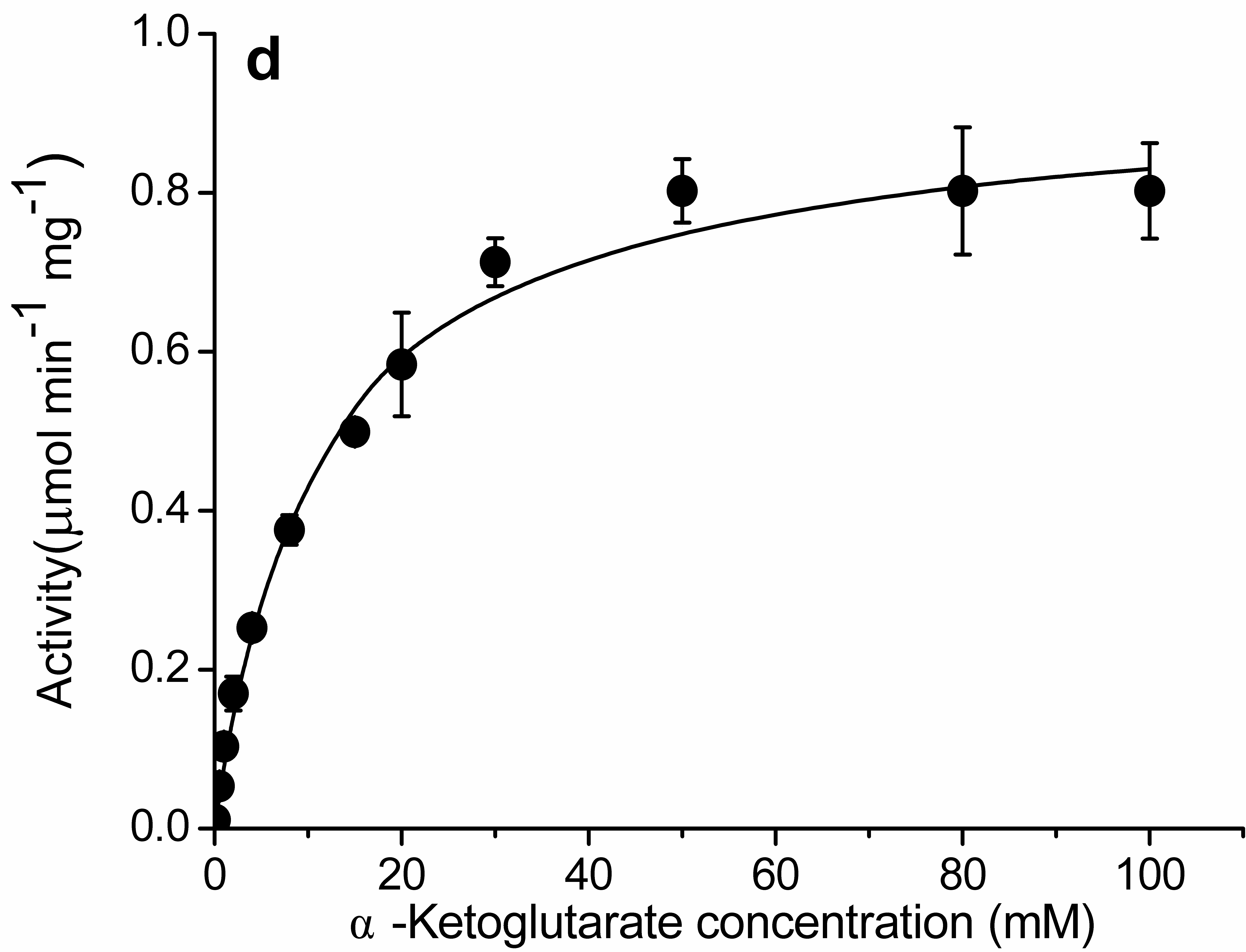


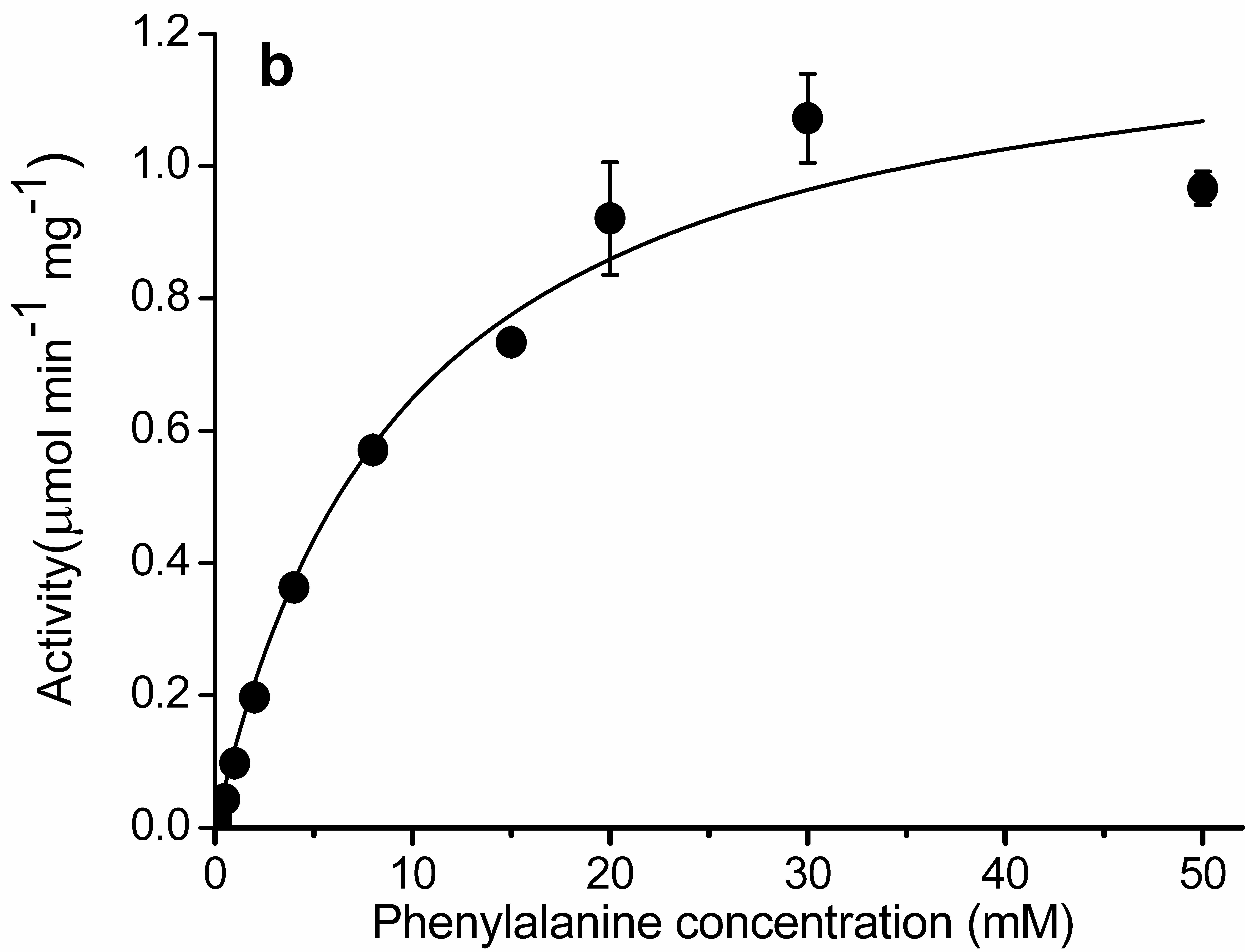

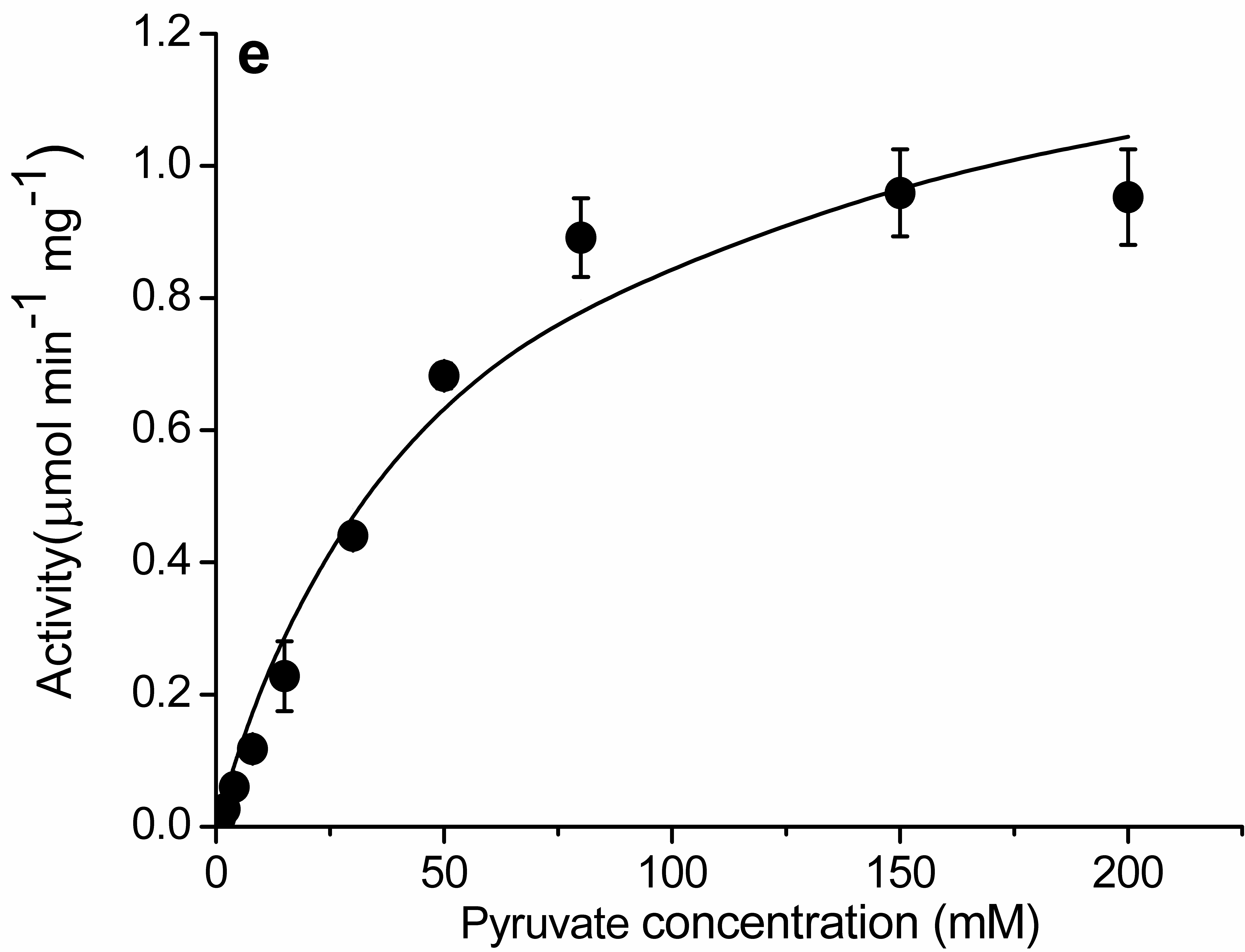


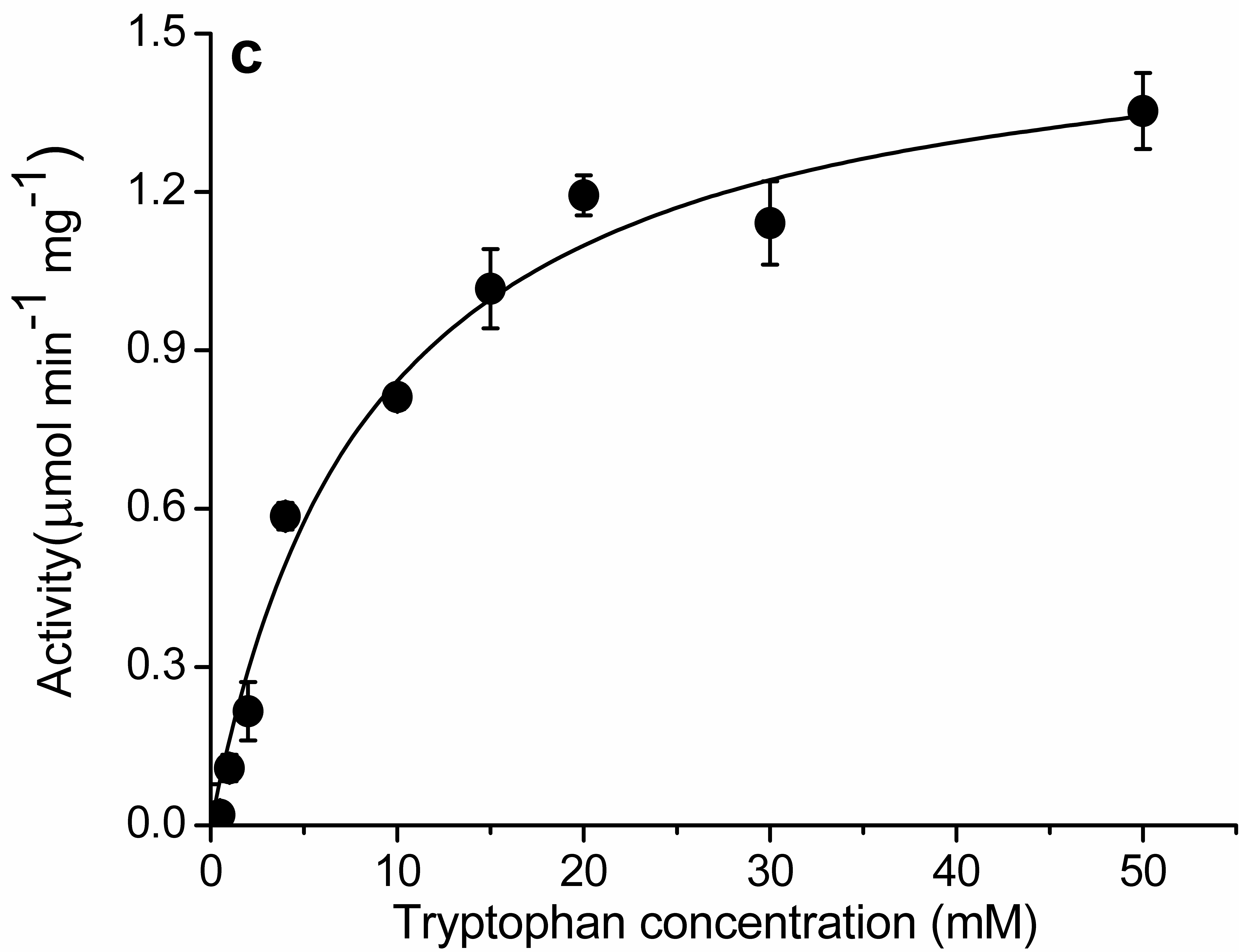

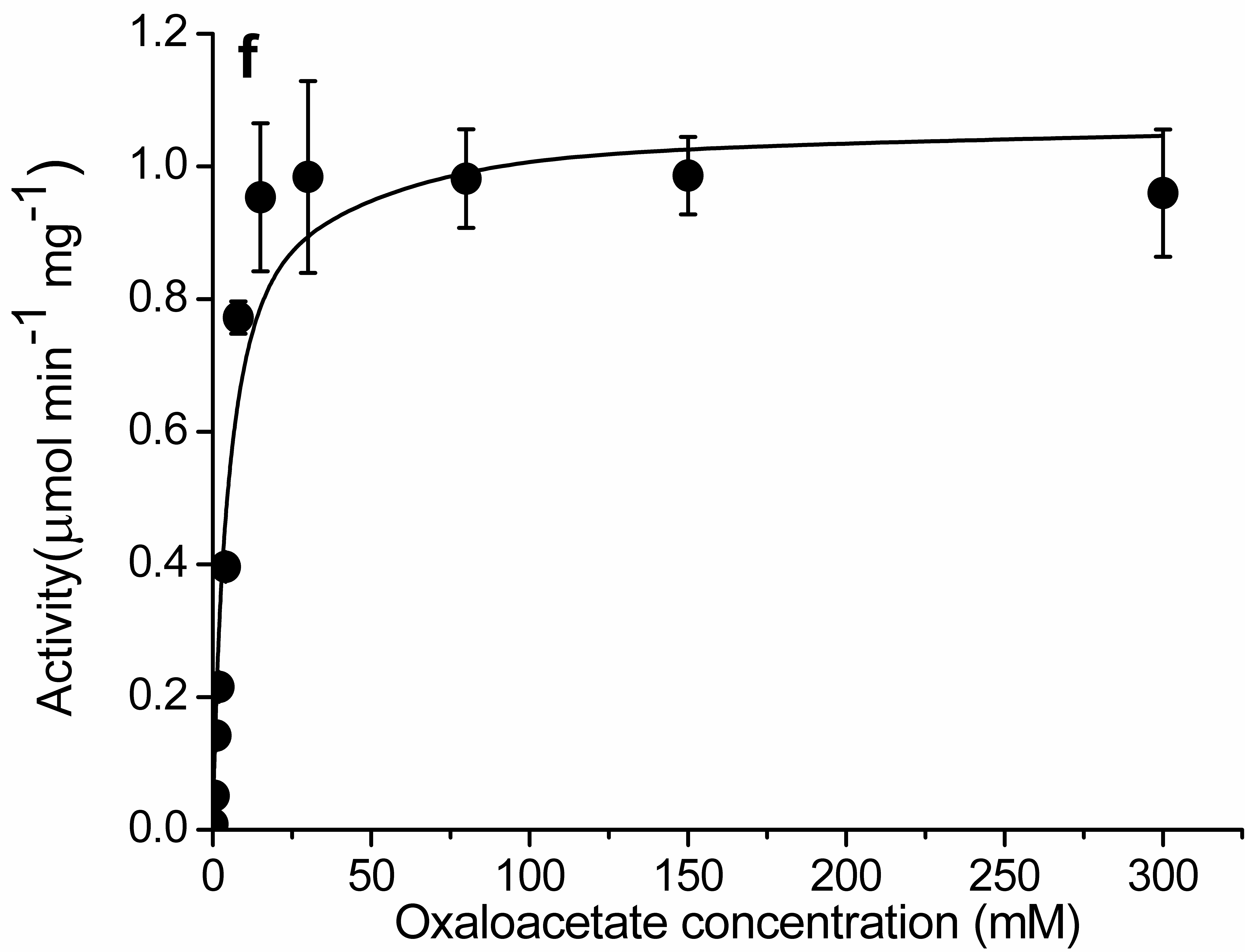


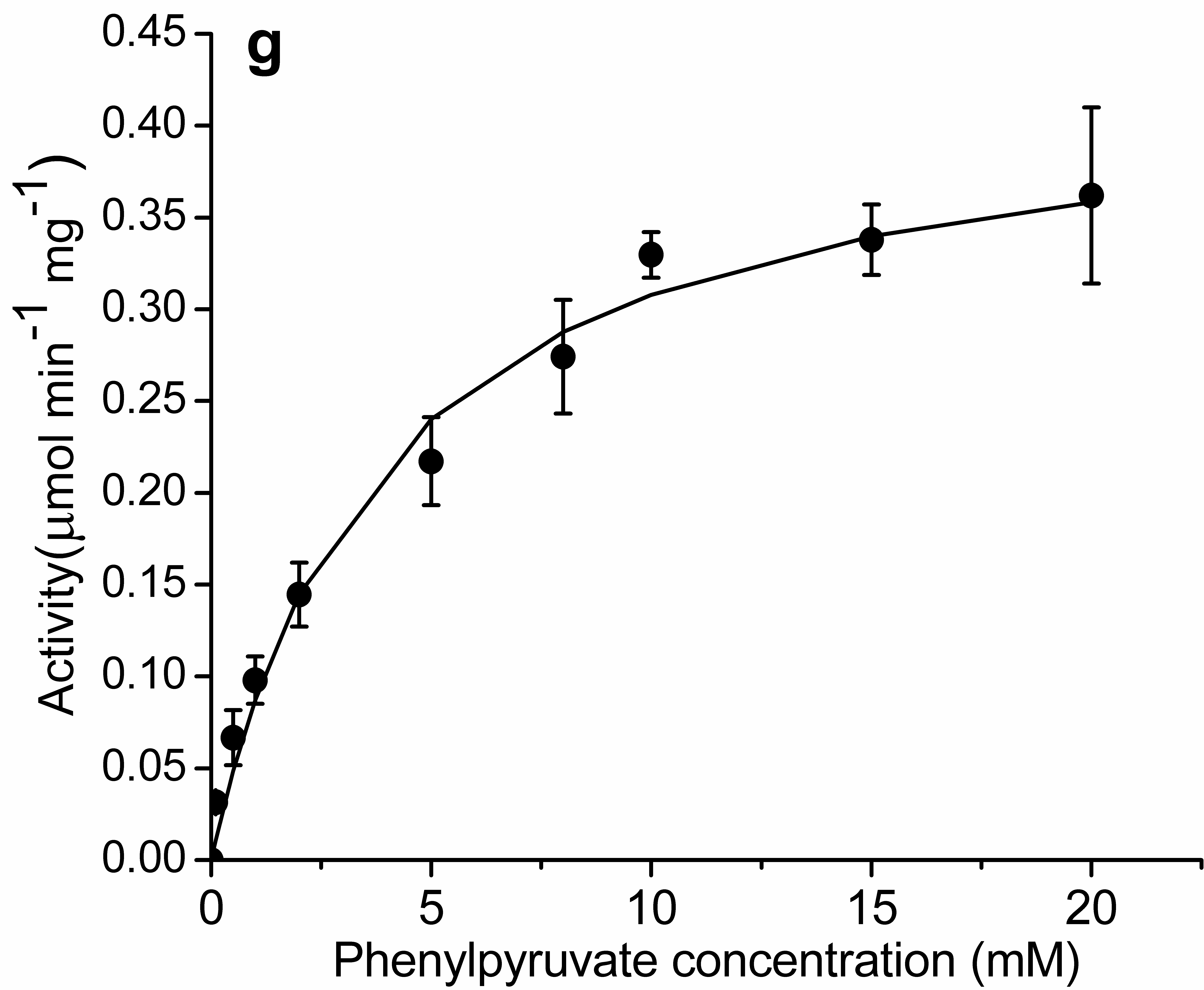

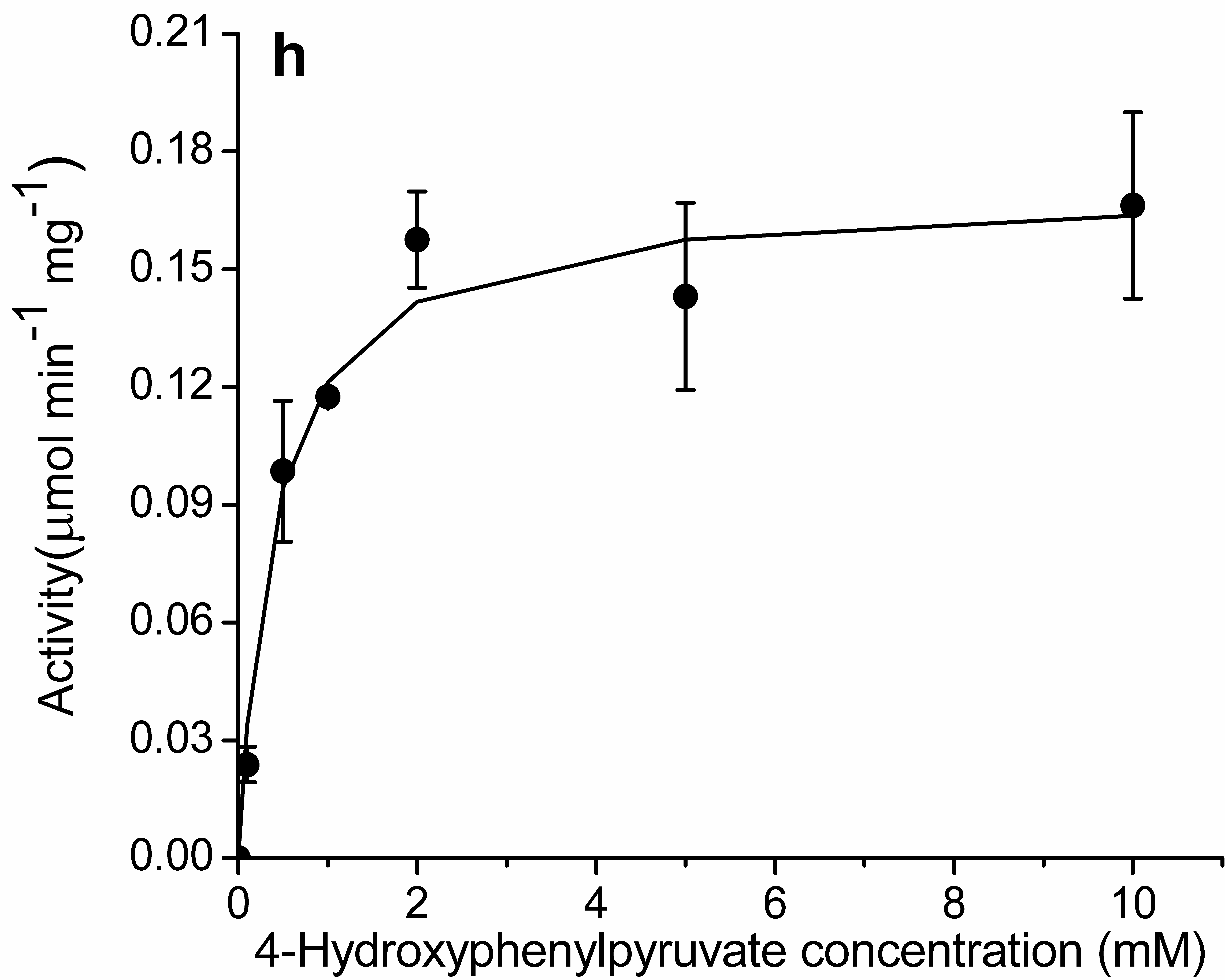


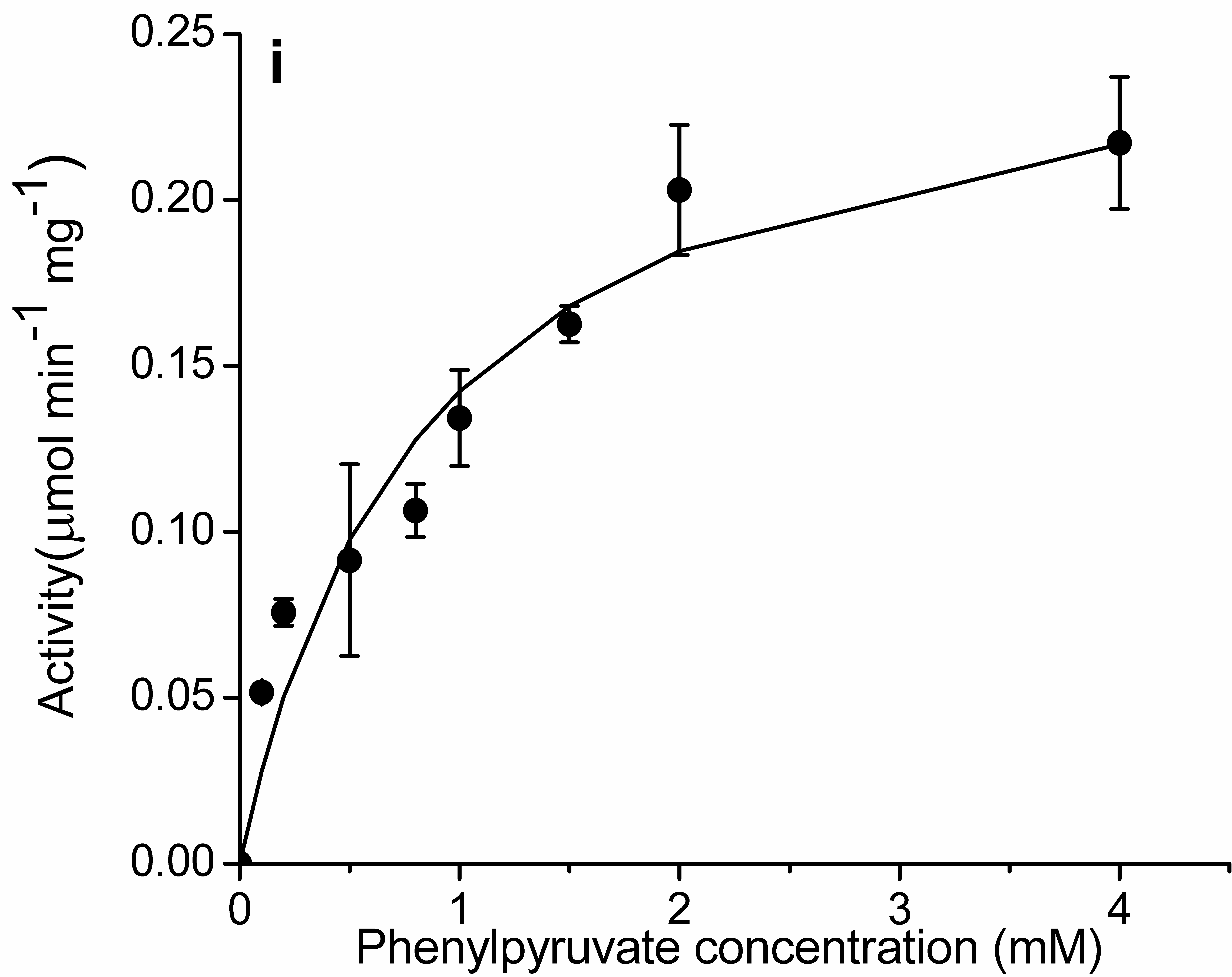

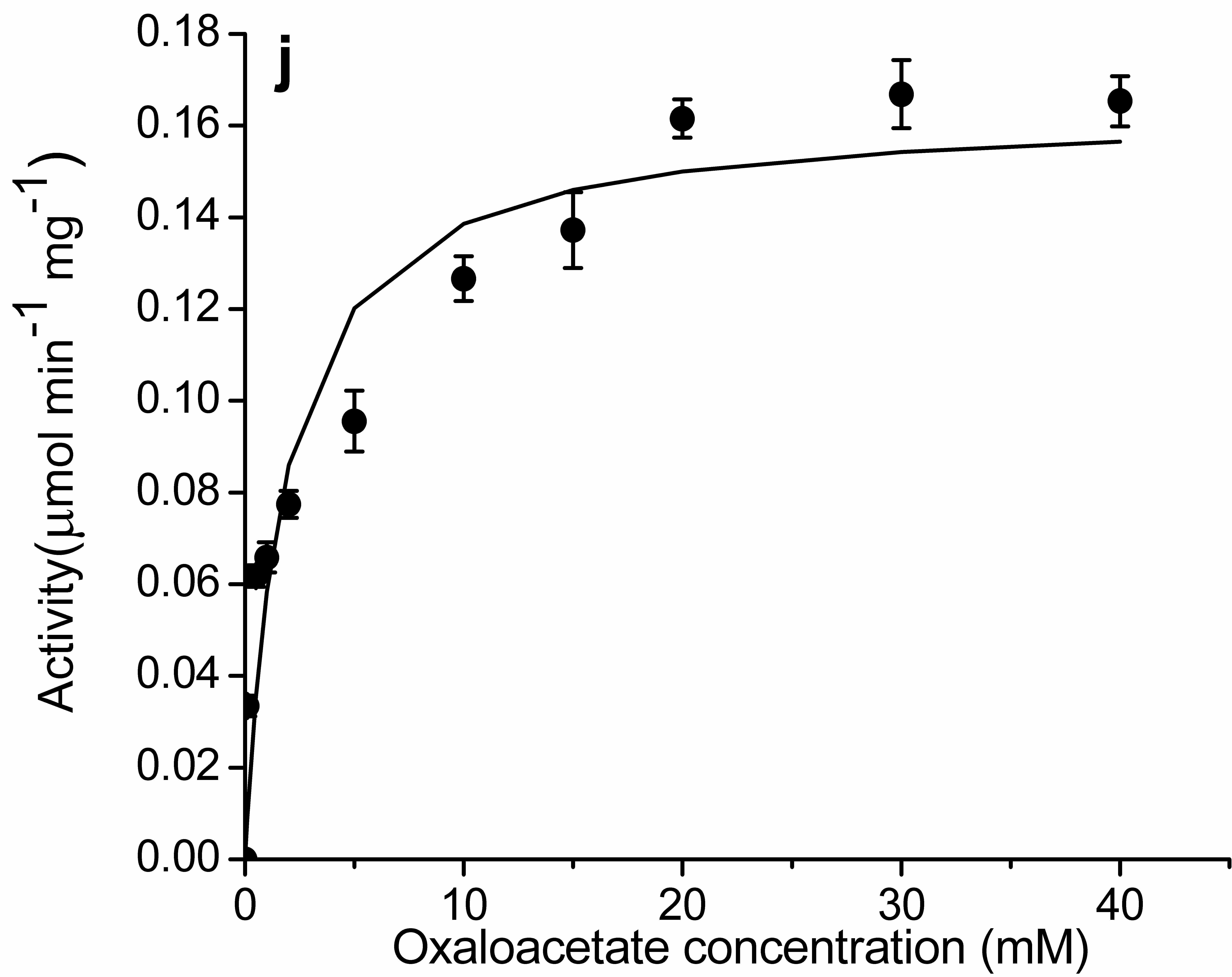


Figure S4. Enzyme kinetics of purified recombinant PvTAT with various substrates at different concentrations. In the forward direction, enzyme kinetics were obtained by monitoring the absorbance of various transamination reaction products 4-HPP at 331 nm, phenylpyruvate at 320 nm, and indole-3-pyruvate at 328 nm corresponding to the substrates L-Tyr, L-Phe, and L-Trp. When α-ketoglutarate (10 mM) was the amino acceptor, 0–4 mM L-Tyr (a), 0–50 mM L-Phe (b), or 0–50 mM L–Trp (c) was used as the amino donor. When L-Tyr (5.5 mM) was the amino donor, 0–100 mM α-ketoglutarate (d), 0–200 mM pyruvate (e), 0–300 mM oxaloacetate (f), or 0–20 mM phenylpyruvate (g) was used as the amino acceptor. In the reverse direction, when L-Glu (10 mM) was the amino donor, 0–10 mM 4-hydroxyphenylpyruvate (h), 0–4 mM phenylpyruvate (i), or 0–40 mM oxaloacetate (j) was used as the amino acceptor. NADH formation was measured at 340 nm using a Multi-Mode Microplate Readers (Spectra Max M 2, Molecular Devices, USA). *V*_max_ and *K*_m_ values were calculated according to a nonlinear regression of the Michaelis-Menten equation, where *V*=(*V*_max_S)/(*K*_m_+S)[^2^](#_ENREF_2). All enzyme assays were performed at an appropriate enzyme concentration so that reaction velocity was linear and proportional to enzyme concentration during the incubation time period. Kinetic data were evaluated by curveExpert 1.4. At least triplicate assays were performed for all data points. Data were represented as means±SD of three replicates.

2 Hernández, A.,Ruiz, M. T. An EXCEL template for calculation of enzyme kinetic parameters by non-linear regression. *Bioinformatics* **14**, 227-228, doi:10.1093/bioinformatics/14.2.227 (1998).


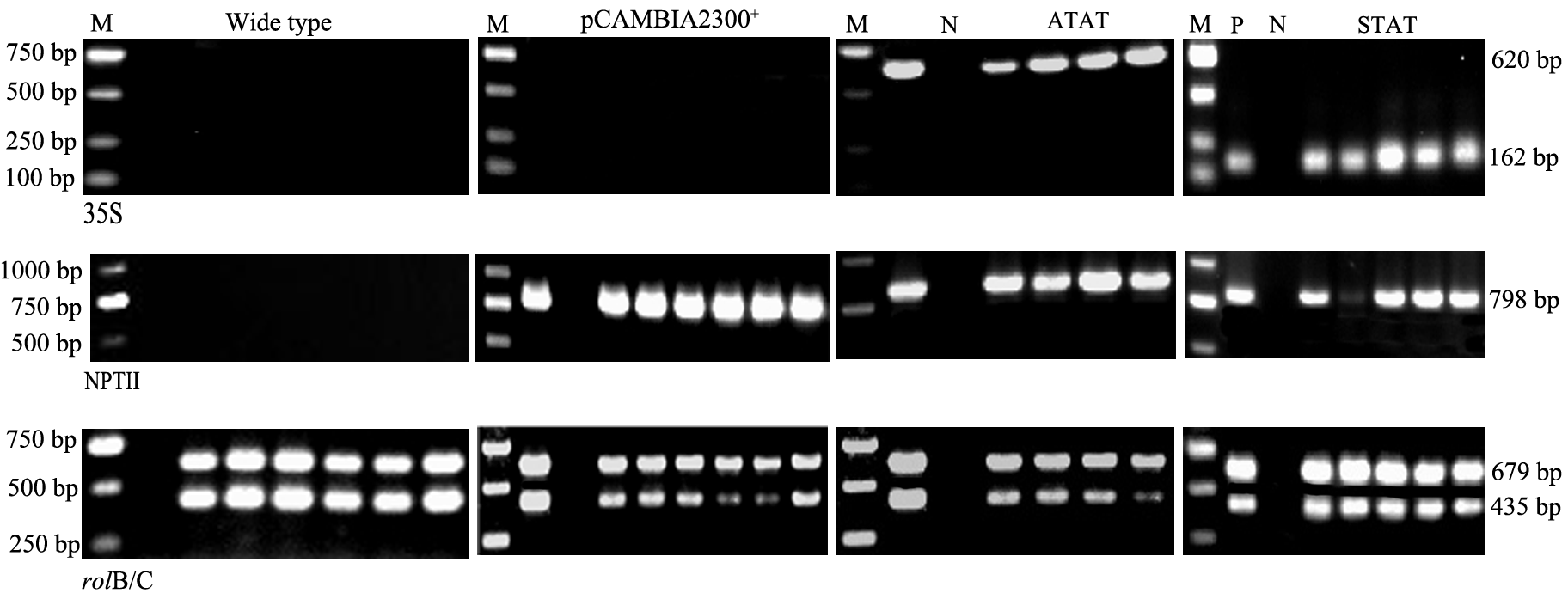


Figure S5. Identification of transgenetic hairy root lines. Four primer pairs, *rol*B/C, *nptII*, 35S forward primer 1(35SF1), and STAT reverse primer (STATR) were designed for the identification of sense-expressed hairy root lines. 35SF was located at the 35S promoter of the pCAMBIA2300 vector, and STAT R was from the *PvTAT* gene. 35S forward primer 2 (35SF2) and ATAT reverse primer (ATATR) were designed for the identification of antisense-expressed hairy root lines. ATAT R was also from the *PvTAT* gene. P, positive control (ATCC 15834 containing the recombinant plasmids). N, negative control (the intact plant).
